# Magnetstein: An Open-Source Tool for Quantitative NMR Mixture Analysis Robust to Low Resolution, Distorted Lineshapes, and Peak Shifts

**DOI:** 10.1021/acs.analchem.3c03594

**Published:** 2023-12-20

**Authors:** Barbara Domżał, Ewa Klaudia Nawrocka, Dariusz Gołowicz, Michał Aleksander Ciach, Błażej Miasojedow, Krzysztof Kazimierczuk, Anna Gambin

**Affiliations:** † Faculty of Mathematics, Informatics and Mechanics, University of Warsaw, Banacha 2, Warsaw 02-097, Poland; ‡ Centre of New Technologies, University of Warsaw, Banacha 2C, Warsaw 02-097, Poland; § Institute of Physical Chemistry, Polish Academy of Sciences, Kasprzaka 44/52, Warsaw 01-224, Poland

## Abstract

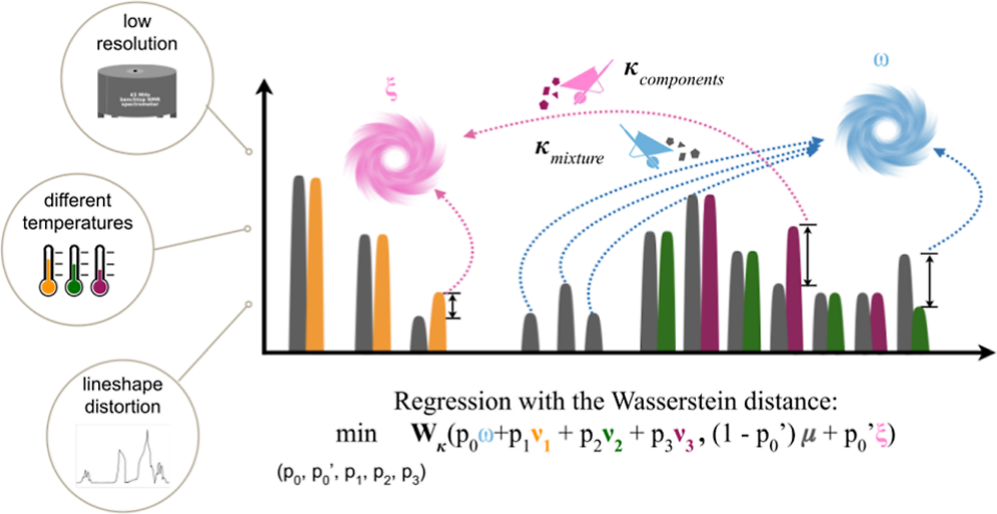

^1^H NMR spectroscopy is a powerful tool for analyzing mixtures including determining the concentrations of individual components. When signals from multiple compounds overlap, this task requires computational solutions. They are typically based on peak-picking and the comparison of obtained peak lists with libraries of individual components. This can fail if peaks are not sufficiently resolved or when peak positions differ between the library and the mixture. In this paper, we present Magnetstein, a quantification algorithm rooted in the optimal transport theory that makes it robust to unexpected frequency shifts and overlapping signals. Thanks to this, Magnetstein can quantitatively analyze difficult spectra with the estimation trueness an order of magnitude higher than that of commercial tools. Furthermore, the method is easier to use than other approaches, having only two parameters with default values applicable to a broad range of experiments and requiring little to no preprocessing of the spectra.

Analytical chemists often deal with mixtures, particularly when studying environmental, industrial, or medical samples. We are usually interested in qualitative and quantitative results, i.e., determining the composition and concentrations of its ingredients. In some tasks, the general classification of a sample is sufficient, e.g., in metabolomic screening, where statistical analysis allows for deciding whether the studied blood or urine comes from a sick or healthy individual. NMR spectroscopy is one of the primary methods for studying mixtures in all the ways mentioned.^1,2^ Being noninvasive and nondestructive, NMR allows the same sample to be tested by other methods or continuously monitored. The nuclear resonance frequencies are sensitive to the molecule’s electronic structure, and thus, an NMR spectrum can serve as a unique chemical fingerprint, allowing qualitative analysis. Quantitative analysis (qNMR) is also possible, and the most reliable way to perform it is to apply the straightforward one-dimensional (1D) pulse-acquire approach. If the interscan delay is sufficiently long compared to the longitudinal relaxation rate, one can calculate concentrations directly from peak intensities in the NMR spectrum, taking into account the number of nuclei contributing to each peak.^3,4^

The 1D spectra, however, often suffer from a peak overlap. The more complex the studied mixture is, the more ambiguities appear in the spectrum, hampering the analysis. Even if compounds can be identified, the integration of overlapping peaks is biased, leading to incorrect concentration estimations. The known remedy to the resolution problem is multidimensional (nD) NMR spectra that resolve peaks in extra dimensions. These can be standard frequency dimensions or other “non-Fourier” dimensions, such as diffusion,^5^ relaxation,^6^ concentration,^7^ or temperature coefficient.^8^ However, the quantitative results are challenging to achieve with nD NMR, primarily due to uneven internuclear coherence transfers and signal losses occurring before acquisition (relaxation during the pulse sequence). Despite many efforts to tackle both problems,^9−12^ the most straightforward pulse-acquire 1D measurements still remain the main workhorse of qNMR. In particular, the 1D ^1^H NMR spectra are employed because they can be acquired quickly with a relatively good sensitivity, a crucial feature for high-throughput applications such as metabolomics.^13^

The peak crowding can be resolved at the spectral processing stage using sophisticated decomposition methods.^14−17^ The obtained peak lists can be compared with spectral libraries to identify compounds in mixtures.^18^ Here, however, we run into another problem—peak shifts dependent on the concentration, pH, ionic strength, and other factors.^19^ Thus, in general, the spectrum of a mixture cannot be considered a simple linear combination of the spectra of individual compounds. More complications arise from differences in sample- and hardware-dependent factors, such as field strength, resolution, and lineshape.

Different approaches have been applied to tackle the problem of the quantification of mixture ingredients.^20−23^ In general, they are based on libraries of spectra and assume that the composition of a sample is either precisely known [as in ACD/Spectrus 2020.1.1 Targeted Profiling tool (Advanced Chemistry Development, Inc., ACD/Labs Ontario, Toronto, ON, Canada)] or that it belongs to a specific class of samples (e.g., a mixture of metabolites). Flexible approaches allow the use of excess libraries that include the compounds [Chenomx,^24^ Mnova (MNova Simple Mixtures Analysis (SMA) and Mnova Automatic NMR Identification and Quantification (MANIQ), Mestrelab Research, S. L., Spain)]. The criteria of comparison with the libraries differ between the various methods. BATMAN^18,22^ uses a Markov Chain Monte Carlo algorithm to fit the mixture spectrum with a combination of idealized spectra of cataloged metabolites and uses wavelets to analyze the remaining signals. AQuA^21,25^ employs the idea of “reporter signals”, specific resonances used for the quantification of specific compounds in the mixture. Dolphin^26^ supports the analysis of crowded 1D spectra with better-resolved 2D data. Other approaches aimed at metabolomic profiling, such as MetaboMiner^27^ or MetaboHunter,^28^ also use sets of 1D and 2D spectra.

Regardless of the computational paradigm employed, all methods have to deal with the mentioned problems of peak overlap and peak shift between a library and a studied mixture. The remedy to the former typically involves peak-picking and deconvolution, which require the assumption of a specific lineshape (usually a Lorentzian/Gaussian). The issue of peak shifts can be solved by allowing horizontal peak drifts by a user-defined margin [as in HMDB search,^29^ MetaboHunter,^28^ MetaboMiner,^27^ and Mnova software (Mestrelab Research, S. L., Spain)].

In this paper, we present an NMR spectral processing method that provides a quantitative analysis and does not rely on peak-picking and deconvolution. This way, it works well for low-field spectra or strong magnetic field inhomogeneity. Processing complex spectra without the need for peak-picking is accomplished by the use of the Wasserstein metric, which can meaningfully compare spectra with different resolutions. Moreover, it is relatively robust to variations in peak positions between the mixture and the library. Our approach is based on finding a combination of library spectra that best fits the mixture spectrum in terms of the Wasserstein metric. We have implemented this approach with an open-source tool called Magnetstein.

## Magnetstein Algorithm

### NMR Spectra as Probability Measures

The standard way of representing NMR spectra is to treat them as vectors of intensity. Here, we offer an alternative mathematical formalism. We treat an NMR spectrum as a discrete probability measure, represented as a finite set of pairs with the first element corresponding to a chemical shift and the second one corresponding to intensity. The intensity of spectrum μ in a point *s* is denoted μ(*s*); if *s* is not a spectral point of μ, then μ(*s*) = 0. The only requirement to represent the spectrum in such a way is to normalize it.

The main difference between our representation and the standard one is the lack of fixed spectral points common for all of the spectra. Therefore, the spectra in the library as well as the mixture may have their own unique chemical shift axes. Moreover, the size and range of the data can vary for different spectra. Still, the operation of adding spectra is well-defined. Provided that *p*_1_ + *p*_2_ +···+ *p*_*k*_ = 1, the sum of spectra ν_1_, ν_2_, ..., ν_*k*_ with proportions *p*_1_, *p*_2_, ..., *p*_*k*_ is also a probability measure. This probability measure, denoted ν_*p*_, is defined as ν_*p*_(*s*_*i*_) = *p*_1_ν_1_(*s*_*i*_) + *p*_2_ν_2_(*s*_*i*_) +···+ *p*_*k*_ν_*k*_(*s*_*i*_) for every spectral point *s*_*i*_ present in any of ν_1_, ν_2_, ..., ν_*k*_. If *s*_*i*_ is not a spectral point for some ν_*j*_, then ν_*j*_(*s*_*i*_) = 0.

We strongly emphasize that representing a spectrum as a probability measure is a solely technical necessity. In fact, we require this representation due to the Wasserstein distance, which is a fundamental concept in our approach and is defined between two probability measures. There is no other underlying rationale for this choice; specifically, we do not assume or suggest any randomness in the spectral points.

### Wasserstein Distance as a Measure of Spectral Similarity

The simplest explanation of the Wasserstein distance (a.k.a. the Earth mover’s distance^30^) envisions the two spectra μ and ν as piles of sand. Intuitively, the Wasserstein distance is the lowest possible effort (in terms of “shoveling”) required to transform the μ-shaped sand pile into the ν-shaped sand pile.^31−35^ The amount of sand transported from point *x* to point *y* is denoted as γ(*x*, *y*), and the function γ is called a transport plan. In the context of NMR, γ(*x*, *y*) denotes the amount of signal transported between points *x* and *y* on the chemical shift axis. The partial effort required to transport the signal from position *x* to position *y* on the chemical shift axis is equal to the distance between the points, |*x* – *y*|, multiplied by the amount of transported signal γ(*x*, *y*). Under a fixed transport plan γ, the total effort to transform μ into ν is the sum of those partial efforts over all points *x* of μ and points *y* of ν. The Wasserstein distance *W*(μ, ν) is defined as the minimum effort over all possible transport plans
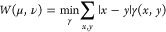


In practice, it can be easily computed in linear time complexity.^31,34^

The Wasserstein distance can meaningfully compare spectra with different line widths. This feature has been successfully exploited in comparing profile mass spectra to centroided ones.^34^ The compared spectra can have different resolutions. Moreover, the Wasserstein distance does not require a perfect agreement of peak positions, and therefore, it can handle slight misalignments between signals of the compared spectra. The only requirement is that the compared spectra are normalized.

### Deconvolution of NMR Spectra

Suppose that we have a library of *k* spectra ν_1_, ν_2_, ..., ν_*k*_ (again, treated as discrete probability measures). Quantification of these components in a spectrum of their mixture μ can be thought of as a problem of finding an optimal linear combination of the components, ν_*p*_ = *p*_1_ν_1_ + *p*_2_ν_2_ +···+ *p*_*k*_ν_*k*_, which approximates μ. Finding such a combination simultaneously solves the problem of quantification and deconvolution because all signals of all components are used. If we use the Wasserstein distance to compare ν_*p*_ and μ, the optimal combination can be found by solving the following problem of linear regression with a special loss function
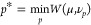
1

However, μ may contain signals from components other than ν_*i*_, e.g., contaminants or background noise, which are not included in the model. Following ideas previously developed for mass spectrometry,^34,35^ we represent such signals by augmenting ν_*p*_ with an auxiliary point ω to which the excess signal can be transported. This leads to an augmented component spectrum *p*_0_ω + ν_*p*_, where *p*_0_ is the proportion of excess signal in μ and 1 – *p*_0_ = *p*_1_ +···+ *p*_*k*_ is the proportion of the signal explained by the library spectra. Setting a denoising penalty κ_mixture_ allows for controlling the amount of signal transported to ω, where removing a fraction *p*_0_ of the signal inflicts an additional cost equal to *p*_0_κ_mixture_.

Just as the spectrum of mixture μ can contain excess signals and noise, so can library spectra ν_*i*_, especially if the library is acquired experimentally rather than predicted theoretically. This is particularly important in the context of NMR, where theoretical predictions are more difficult than, e.g., in mass spectrometry. Therefore, to adapt the regression method to NMR, we introduce a second auxiliary point ξ, which gathers the excess signals from the library with a penalty κ_components_. This leads to an augmented mixture spectrum *p*_0_^′^ξ + (1 – *p*_0_^′^)μ.

The task is now to simultaneously estimate the proportions *p*_1_, ..., *p*_*k*_ and find which signals need to be transported to ω (i.e., removed from μ and labeled as unexplained by the library) and which signals need to be transported to ξ (i.e., removed from the library and labeled as missing in μ). This can be expressed succinctly as minimizing the Wasserstein distance between the augmented mixture spectrum *p*_0_^′^ξ + (1 – *p*_0_^′^)μ and the augmented components spectrum *p*_0_ω + ν_*p*_
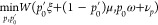
2

As proved in the Supporting Information, problem (2) can be formulated as a linear program and then converted to a sparse dual form. We used this formulation to implement an algorithm to solve this problem in the open-source Python 3 package. The problem’s sparsity allowed us to optimize the algorithm with respect to the time of computation and memory usage. The core of the algorithm, i.e., the dual linear program, is solved via a simplex method implemented in the Gurobi Optimizer.^36^

### Interpretation of the Denoising Penalties

**Figure 1:**
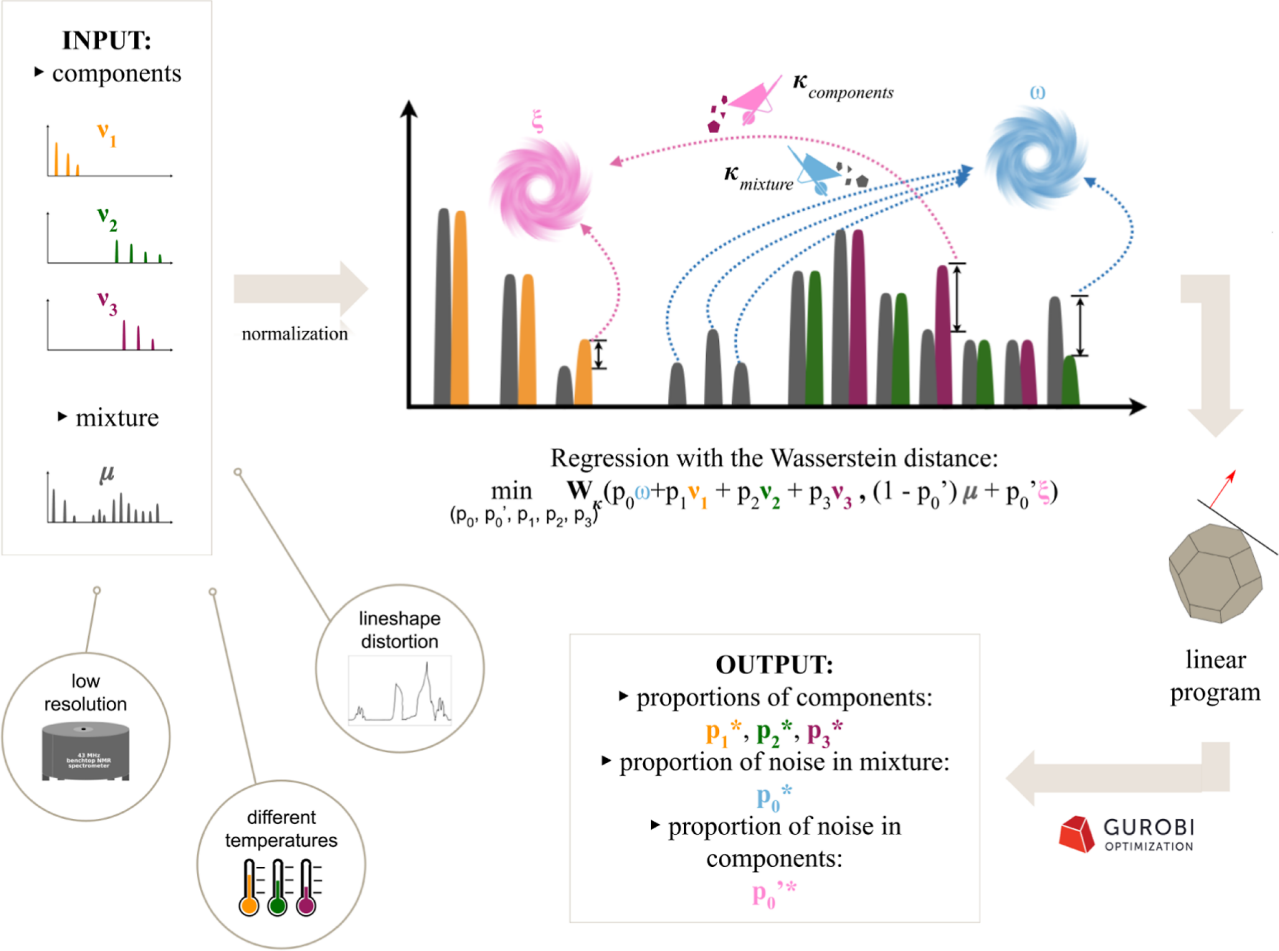
Scheme of the Magnetstein workflow. The input comprises a library of component spectra ν_1_ (yellow), ν_2_ (green), ν_3_ (purple), and a mixture spectrum μ (gray), treated as discrete probability measures (see Section NMR Spectra as Probability Measures). The central part of the scheme visualizes the flow of the signal under the optimal transport plan. We can imagine the optimization process as rescaling the components’ spectra in such a way that the scales sum up to one and that they minimize the Wasserstein distance between the mixture’s spectrum and the components’ spectra added together after rescaling. As dynamic scale manipulations cannot be represented in a static scheme, the heights of the components’ peaks reflect the resulting optimal proportions of components. The optimal transport plan is as follows. The two left-most yellow peaks are transported to the two left-most gray peaks. The situation is analogous to the three left-most green peaks and the two outer purple peaks. Meanwhile, the right-most yellow peak is higher than the corresponding gray peak, so its protruding part is assigned to an auxiliary point ξ designated for excess signals present in the components’ spectra. The same occurs with the middle purple peak. On the other hand, the three gray peaks in the middle of the spectrum have no counterparts in the components’ spectra, so they are transported to a second auxiliary point ω, designated for excess signals in the mixture’s spectrum. Lastly, the right-most gray peak, which is higher than its green counterpart, is split under the transport plan: a part of it is transported to the right-most green peak, and another part lands in the second auxiliary point ω. For the sake of computational efficiency, the optimization problem here is reduced to a linear program and solved by the simplex algorithm using Gurobi.^36^ The method is robust to differences in peak positions and in lineshapes between ν_*i*_ and μ caused by low-resolution and varying experimental conditions.

The auxiliary spectra ω and ξ can be interpreted as points located outside of the chemical shift axis, equidistant from any resonance frequency value, with distances κ_mixture_ and κ_components_, respectively. This interpretation of ξ and ω is shown in Figure 1, with the auxiliary points represented as vortexes. Now, if a given signal in ν_*p*_ is further away than κ_components_ from any observed signal in μ, then ξ is the closest point to which this signal can be transported, and thus, the optimal transport plan prefers to remove it from ν_*p*_ rather than match it with μ. Symmetrically, if a given signal in μ is further away than κ_mixture_ from any signal in ν_*p*_, removing this signal by transporting it to ω inflicts a partial cost lower than that matching it with ν_*p*_. Therefore, both denoising penalties can be interpreted as the maximum expected difference in resonance frequencies between signals from the library and the mixture spectrum.

However, the denoising penalties are not equivalent to simply matching peaks within predefined bins. This is because the optimization problem (2) considers the overall distribution of the signal in the whole spectrum when deciding whether to remove a given signal. This allows for more flexibility in setting the denoising penalties, which in turn means that a single set of values is applicable for a broader range of experiments.

## Experimental Section

### NMR Spectroscopy

We prepared samples representing different challenges of mixture analysis and acquired their ^1^H NMR spectra. We list the five main challenges discussed in our study in Table 1. Additionally, we conducted four extra experiments, which we list in Table S1 (Experiments 6–9). As required by our approach, we also prepared a library by collecting individual ^1^H NMR spectra for all components of our samples. Finally, the method has been tested on artificial mixtures of three amino acids at different relative concentrations (Experiment 10) and an authentic food product involving them: the essential amino acid supplement (Experiment 11).

**Table 1:** Results of Estimation for Magnetstein with Constant, Fixed Values of Parameters (κ_mixture_ = 0.25, κ_components_ = 0.22), for ACD/Labs’ ACD/TP, and for Mestrelab’s MANIQ^a^

			ingredients’ proportions			
exp	problem	ingredients	real	Magnetstein	ACD/TP	MANIQ
1	large intensity differences between peaks	α-pinene	0.09	0.09	0.06	0.11
		benzyl benzoate (in CDCl_3_)	0.91	0.91	0.94	0.89
2	peak overlap and contamination (extra peaks)	limonene	0.50	0.48	0.46	0.49
		α-pinene (in CDCl_3_)	0.51	0.52	0.54	0.51
3	low resolution (43 MHz benchtop NMR spectrometer)	benzyl benzoate	0.11	0.11	0.11	0.11
		isopropyl myristate	0.73	0.75	0.71	0.76
		limonene	0.09	0.06	0	0.10
		α-pinene	0.08	0.08	0.18	0.00
4	lineshape distortion (shim z1 and z2)	lactate	0.30	0.32	0.35	0.33
		alanine	0.22	0.21	0.28	0.27
		creatine	0.13	0.13	0.19	0
		creatinine	0.20	0.20	0.01	0.27
		choline chloride (in D_2_O)	0.15	0.14	0.17	0.16
5	peak position mismatch between library and mixture (different temperatures)	lactate	0.30	0.32	0.31	0.28
		alanine	0.22	0.21	0.24	0.22
		creatine	0.13	0.13	0.00	0.14
		creatinine	0.20	0.20	0.32	0.23
		choline chloride (in D_2_O)	0.15	0.14	0.14	0.14

a Computation times for Magnetstein ranging from 5 s (Exp. 3) to 171 s (Experiment 1) on the 11th Gen Intel Core i7-11850H@2.50 GHz × 16 processor. “Real” proportions are calculated based on integrals of well-separated peaks from high-field NMR measurements. The integration was performed using Mnova software.

For Experiments 1–2 and 4–9, we acquired the data using a high-field Agilent 700 MHz DirectDrive2 NMR spectrometer equipped with a room-temperature HCN probe at 22.27 °C (Experiments 1, 2, 4, and 6–9) and 36.7 °C (Experiment 5). We calibrated the temperature using an ethylene glycol sample. For Experiments 1, 2, and 6–8, we performed the ^1^H NMR experiments with the following acquisition parameters: 64k data points, an acquisition time of 2.94 s, and one transient. For Experiments 4, 5, and 9, we acquired ^1^H NMR spectra with water-suppression (PRESAT pulse sequence) using the following parameters: 64k data points, an acquisition time of 2.94 s, a relaxation delay of 60 s including 30 s of solvent signal presaturation with a saturation power of −12 dB, and four transients.

We collected ^1^H NMR data for the mixture and ingredients from Experiment 3 on a Magritek Carbon 43 MHz benchtop NMR spectrometer at approximately 28 °C using Spinsolve Expert software. We used the following acquisition parameters: 16 K data points, an acquisition time of 3.28 s, a relaxation delay of 16.72 s, and 1 transient (library) and 32 (mixture).

We also collected spectra on a benchtop NMR spectrometer for the mixtures composed of three branched-chain amino acids (BCAA), namely, leucine, isoleucine, and valine. We collected four spectra for the self-prepared mixtures of leucine, isoleucine, and valine in D_2_O at different molar proportions: (a) 0.34:0.33:0.33, (b) 0.25:0.25:0.5, (c) 0.25:0.49:0.25, and (d) 0.21:0.39:0.40. We refer to this example of four self-prepared BCAA mixtures as Experiment 10. Additionally, we prepared the BCAA mixture by dissolving a food supplement product (instant BCAA powder by Sports Supplements Limited t/a Bulk, a company registered in England and Wales) in D_2_O. The real molar proportions of Leu, Ile, and Val in the solution were 0.48:0.24:0.28, respectively. We refer to this real product example as Experiment 11. We collected all ^1^H NMR spectra for BCAA mixtures (including spectra for the library) with 16,000 data points, an acquisition time of 3.28 s, a relaxation delay of 16.72 s, and 256 transients. We calculated the real proportions of the ingredients in the food supplement product and self-prepared BCAA mixtures by integrating well-resolved peaks in the complementary high-field ^1^H NMR experiments.

### Spectral Analysis

We compared Magnetstein’s performance with that of two other modern software packages designed to solve the mixture analysis problems, i.e., the Gears MANIQ 1.0 plugin for Mnova 14.3.1 software (Mestrelab Research, S. L., Spain) and the ACD/Spectrus 2020.1.1 Targeted Profiling tool (Advanced Chemistry Development, Inc., ACD/Labs Ontario, Toronto, ON, Canada). The packages are referred to in the text as MANIQ and ACD/TP.

In order to run mixture composition analysis in MANIQ, we processed the data in Mnova 14.3.1 software (Mestrelab Research, S. L., Spain): we zero-filled data to 128k (Experiments 1–2 and 4–9) and 64k points (Experiment 3), applied an exponential weighting function (0.1 Hz line broadening for Experiments 1–2 and 4–9 and 0.2 Hz for Experiment 3), corrected phase, and corrected baseline using the polynomial fit (12th order).

Analogously, using the same parameters, we processed data for the analysis in the ACD/Spectrus 2020.1.1 Targeted Profiling tool using the ACD/Spectrus Processor 2020.1.1 NMR Workbook Suite (Advanced Chemistry Development, Inc., ACD/Labs Ontario, Toronto, ON, Canada).

We aligned the spectra to the solvent peak (7.25 ppm (CDCl_3_) for Exp. nos. 1, 2, 6, 7, and 8 (ingredients), 4.63 ppm (D_2_O) for Exp. nos. 4, 5, and 9, and isopropyl myristate for Exp. no. 3).

Parameters for identification and quantification in the ACD/Spectrus Targeted Profiling tool (Advanced Chemistry Development, Inc., ACD/Laboratories Ontario, Toronto, ON, Canada) for all experiments were default. For the Gears MANIQ 1.0 plugin for Mnova 14.3.1 software (Mestrelab Research, S. L., Spain), the parameters of identification for Experiment nos. 1, 2, 4, and 6–9 were default chemical shift tolerance: 0.2000 ppm; labile chemical shift tolerance: 0.4000 ppm; FM match: 0.25; NP match: 0.90; minimum score 0.00; filter threshold: 0.50; and limit: 16 compounds and for Experiment nos. 3, 5, and 8 were default chemical shift tolerance: 2.0000 ppm (had to be increased 10× due to significant peak overlap in Exp. 3 and position mismatches in Exps. 5 and 8); labile chemical shift tolerance: 2.0000 ppm; FM match: 0.25; NP match: 0.90; minimum score 0.00; filter threshold: 0.50; and limit: 16 compounds. The *f* factor method for quantitative calculations in all experiments was set at default.

## Results and Discussion

**Figure 2:**
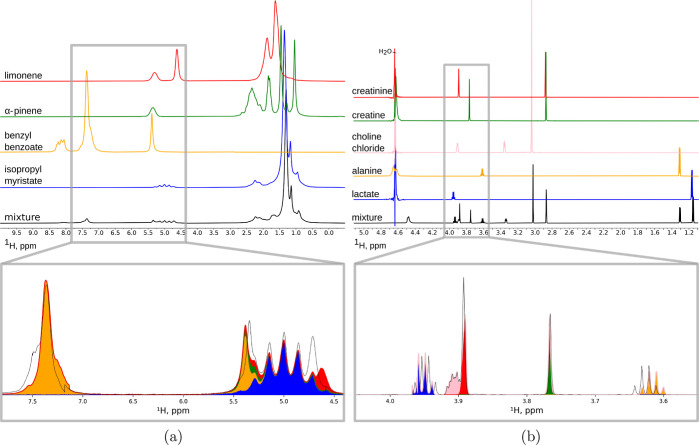
Input spectra of individual components and the mixture for Experiment 3 [(a) top] and Experiment 5 [(b) top] and components added in proportions estimated by Magnetstein together with the mixture’s spectrum for Experiment 3 [(a) bottom] and Experiment 5 [(b) bottom]. The sum of components and the mixture’s spectrum do not ideally fit to one another because of peak shifting. This effect is detected and neutralized by Magnetstein, which nevertheless returns the correct estimation; see Table 1 for details.

The quantitative mixture analysis by NMR is associated with numerous pitfalls. We designed our test experiments to reproduce the most common cases of problematic mixture spectra and show how Magnetstein and two state-of-the-art programs (MANIQ and ACD/Spectrus-Target-Protected Profiling) can deal with them. We assess the trueness of a program with the averaged relative error AvRE, where RE = |*p*_estimated_ – *p*_true_|/*p*_true_. The detailed results are gathered in Tables 1 and S1. The spectra and the output from Magnetstein are shown in Figures 2 and S1. Unless otherwise stated, all programs have been run with the default settings. Later, we discuss how and when the Magnetstein parameters (κ_mixture_ and κ_components_) can be tuned.

### Improved Quantification of Low-Concentration Analytes

**Figure 3:**
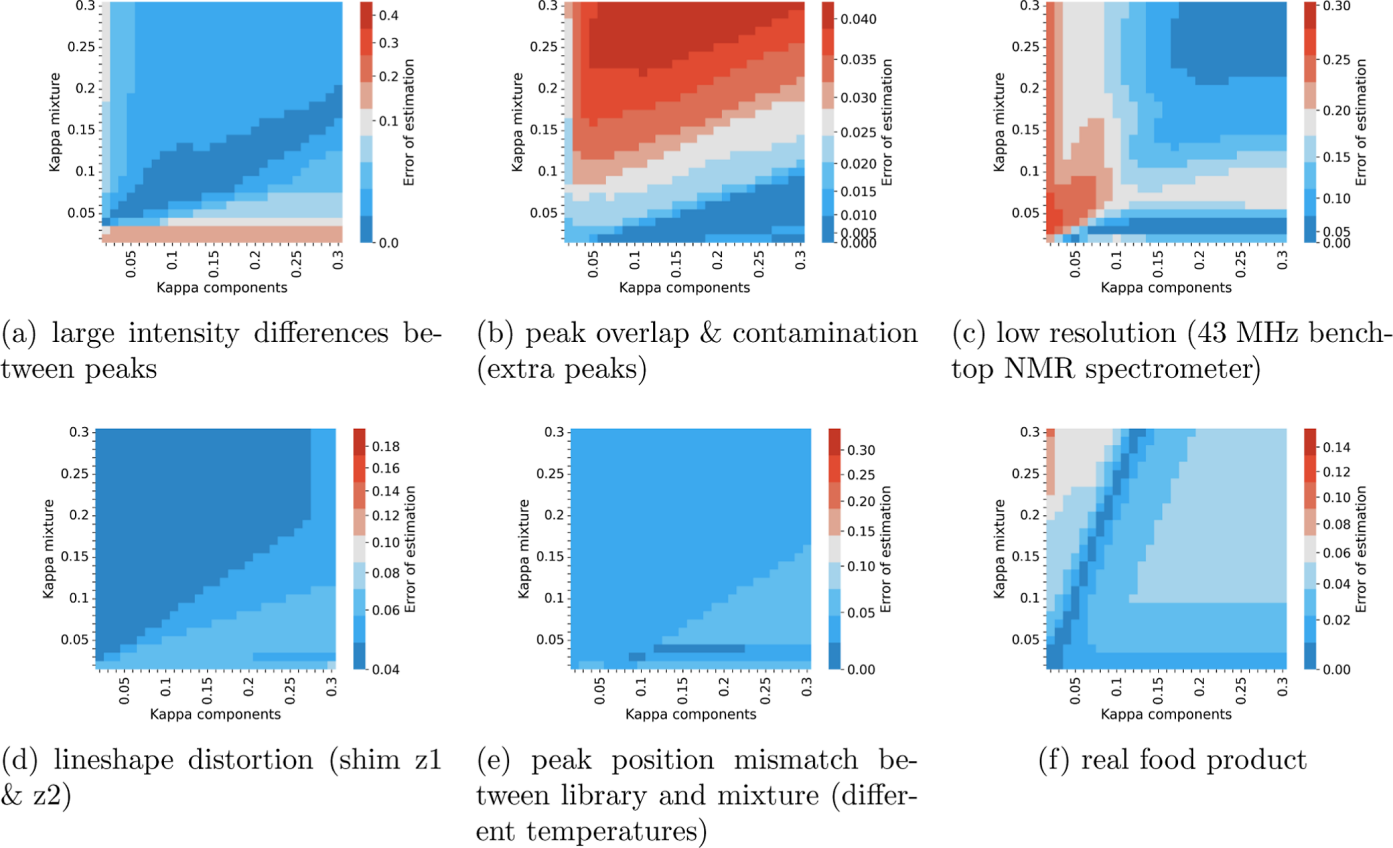
Error of estimation for different values of κ_mixture_ and κ_components_ parameters for experiments 1 (a), 2 (b), 3 (c), 4 (d), 5 (e), and 11 (f). The error is defined as the sum of the absolute differences between the true value of proportion and the estimated value of proportion over all of the components. For the default values of parameters (i.e., κ_mixture_ = 0.25, κ_components_ = 0.22), the proportion of noise in a spectrum (*p*_0_) of a mixture is estimated as 0.0101, 0.0037, 0.0203, 0.0000, 0.0000, and 0.0241 for experiments 1–5 and 11, respectively. The proportion of noise in a linear combination of components (*p*_0_^′^) is estimated as 0.0137, 0.0156, 0.0185, 0.0119, 0.0140, and 0.0000 for experiments 1–5 and 11, respectively.

We started our studies with a relatively straightforward case of a two-component mixture (Experiment 1, α-pinene and benzyl benzoate). The peaks in the spectra were well separated, and the sensitivity was high. However, the concentration difference between the two components was quite significant (ca. 1:10). Magnetstein estimated the relative concentrations with a very high trueness (AvRE 0.0%), an order of magnitude better than that of the other two programs (AvRE 18.3% for ACD/TP, 12.2% for MANIQ), which struggled with the low-concentration compound. The results of Magnetstein were stable for a broad range of values of κ_components_ and κ_mixture_ (Figure 3a). However, for small values of κ_mixture_, the method tends to put the smaller component into vortex ω, causing a large relative error. Therefore, if the goal is to analyze low-concentration compounds, then κ_mixture_ needs to be sufficiently high.

It is noteworthy that Magnetstein can use an external standard for quantification. To do this, one has to acquire a quantitative spectrum of a known substance at a known concentration and algebraically add it to the spectrum of a studied mixture. If the same reference spectrum is used in the library, the absolute concentrations of the mixture components can be calculated from the obtained proportions *p*_*i*_.

### Robustness to Contaminating Signals

As a second example, we considered a two-component mixture with overlapping peaks and low-intensity extra resonances from products of degradation (Experiment 2: limonene and α-pinene). All three methods dealt quite well with the problem, with the best results provided by MANIQ (AvRE 3.0% for Magnetstein, 6.9% for ACD/TP, and 1.0% for MANIQ). In this case, Magnetstein’s performance can be greatly improved by changing the default κ_mixture_ (see Figure 3b). However, the direction of change is opposite to that in Experiment 1. This is because the contaminating peaks, which are the main source of error in this case, are removed when κ_mixture_ is sufficiently small. This example illustrates the trade-off in setting κ_mixture_, where lower values are useful to remove contaminations, while higher values help to estimate low-concentration analytes.

### Robustness to Signal Overlaps

Experiment 3 represents a significantly more difficult situation than the first two. Here, we tested a mixture of compounds used in the fragrance industry. The spectra have been collected on a low-field benchtop spectrometer, a kind of NMR instrument accessible to even small industrial companies. As can be seen in Figure 2a, the resolution is so low that the majority of peaks overlap almost completely. As a consequence, both algorithms based on peak-picking, i.e., ACD/TP and MANIQ, fail to estimate the concentrations of α-pinene and/or limonene (AvRE = 56.9%, 28.8% for ACD/TP, MANIQ; RE for α-pinene = 125.0%, 100.0%; RE for limonene = 100.0%, 11.1%). Magnetstein, on the other hand, still performs well (AvRE = 9.0%; RE for α-pinene = 0.0%; RE for limonene = 33.3%). This can be explained by the fact that our method does not operate on peak lists and thus is not affected by the accuracy of the peak-picking. Notably, the method is very robust to the setting of κ_mixture_, and the value of κ_components_ plays a bigger role (Figure 3c). This can be explained by a high signal-to-noise ratio, so that denoising of the mixture spectrum is less important for accurate quantification. Note that the best results are obtained when κ_components_ is higher than κ_mixture_.

The second example of analysis performed on a spectrum with overlapping peaks is presented in the Supporting Information (Experiment 7).

### Robustness to Lineshape Distortions

Experiment 4 is an example of a mixture spectrum acquired in an inhomogeneous magnetic field (the reference spectra of separated components were well-shimmed). As in the benchtop case, the use of the Wasserstein metric and the lack of peak-picking steps in Magnetstein make it very robust to lineshape distortions (AvRE 3.6%), while peak-based methods struggle (AvRE 39.7% for ACD/TP, 34.9% for MANIQ). In this case, the results are particularly stable for different settings of κ_mixture_ and κ_components_ (Figure 3d), with a low error for all inspected values. This is because, in this example, the errors of estimation are caused by line-shape distortions rather than contaminating signals, and the Wasserstein metric is robust against such distortions.

The Supporting Information shows another example of an analysis of a wrongly shimmed spectrum (Example 9).

### Robustness to Peak Shifts

In Experiment 5, we attempted to mimic the problem of peak shifts between the library and the mixture, which is common in metabolomic studies. Figure 2b shows the spectra of common metabolites acquired at 22.27 °C and the spectrum of their mixture acquired at 36.7 °C. As can be seen, the peak shifts are significant. Still, Magnetstein achieves a high trueness (AvRE = 3.6%) and outperforms other methods (AvRE = 35.8, 7.2% for ACD/TP, MANIQ). Good results are obtained for all of the inspected settings of κ_mixture_ and κ_components_ (Figure 3e), again owing to the intrinsic robustness of the Wasserstein metric against peak shifts.

The Supporting Information shows another example of an analysis of the spectrum with peaks shifted compared to those of the library, this time due to the use of different solvents (Example 8).

The results of the Magnetstein calculations gathered in Table 1 were obtained for the default settings of κ_mixture_ and κ_components_. As seen in the heatmaps in Figure 3a–e, while the default parameters provide a good estimation for diverse experiments, the optimal parameters depend on the problem at hand. Therefore, if possible, it is recommended to tune the parameters using a calibration mixture with a known composition. In Table S1, we show the results of our experiments obtained with optimal κ_mixture_ and κ_components_.

### Dietary Supplement Composition Analysis

After carefully evaluating Magnetstein’s performance versus that of other popular tools, we applied our method to study the composition of a dietary supplement product (see Experiment 11 in the Experimental Section for details). Magnetstein allowed us to estimate the proportions of the three supplement ingredients as 0.50(4.2%) for leucine, 0.25(4.2%) for lsoleucine, and 0.25(10.7%) for valine. The numbers in parentheses indicate the relative error. Except for analyzing this commercially available product, we studied several self-prepared mixtures of leucine, isoleucine, and valine at various concentrations that mimicked potential variations of this product (see Experiment 10 in the Experimental Section for details). As for the four studied mixtures, we obtained proportions of (a) 0.36(5.9%), 0.34(3.0%), 0.30(9.1%); (b) 0.28(12.0%), 0.22(12.0%), 0.50(0.0%); (c) 0.28(12.0%), 0.48(2.0%), 0.25(0.0%); and (d) 0.25(19.0%), 0.34(12.8%), 0.41(2.5%). Considering the very high complexity of the spectrum (see Figure S12), where peaks from different ingredients are highly overlapped, we think these results are satisfactory. We believe benchtop NMR spectroscopy assisted by Magnetstein can potentially become a fast and cost-efficient testing tool for dietary supplements and similar products.

One question that naturally arises is whether it is possible to generalize Magnetstein to higher-dimensional data. It turns out that this task is not straightforward. The effective linear program representation that we provide in the Supporting Information is strictly associated with one-dimensional spectra. In particular, Theorem 2 from Supporting Information does not have its higher-dimensional equivalent. Therefore, the problem of computing the Wasserstein distance between two- and more-dimensional objects is much more computationally demanding. We are aware of two approaches that could be useful in this case. The first one is called the sliced Wasserstein distance and is based on computing several projections to one-dimensional space.^37^ The second one involves using a graph theory formalism to represent signal transport as flows in a network. Our plan is to investigate the usability of these ideas in higher-dimensional problems in our further research.

## Conclusions

We have presented a conceptually new approach to quantitative NMR analysis of mixtures and demonstrated its advantages over the conventional approach. The latter is to compare lists of peaks between the mixture and library spectra. This approach is sensitive to peak shifts and lineshape distortions, often resulting in inaccurate quantification. In contrast, we consider spectra as probability distributions of signal intensity and use the mathematical tools of the optimal transport theory. This allows for precise quantification even in the presence of considerable contaminations, lineshape distortions, peak shifts, and low resolution. In these more difficult cases, our open-source tool improves the quantification by orders of magnitude compared with state-of-the-art commercial solutions. A careful mathematical modeling of the quantification problem allowed for creating a tool that requires minimal preprocessing of the data and relies only on two parameters, which are flexible enough that a single set of values is applicable to a broad range of experiments.

The probabilistic interpretation unifies low- and high-resolution spectra in a single mathematical formalism. In particular, one could use different measurement conditions to acquire libraries and spectra of mixtures. This opens the possibility to further develop computational methods for benchtop NMR instruments, which are readily available for many laboratories and useful for rapid screening of samples, but their low resolution poses a major obstacle in more complex analyses. Furthermore, the probabilistic paradigm is applicable for any kind of spectroscopy where the signal is non-negative and spectra can be approximated as linear combinations; in fact, computational methods based on the Wasserstein distance, including a simpler version of the Wasserstein regression, have already been shown to provide accurate results in mass spectrometry.^34,35,38^ While each spectroscopic technique has its own specifics that need to be considered, Magnetstein has the potential to be a single tool that unifies the quantitative analyses of multiple kinds of spectroscopic data.

## Data Availability

The data are available at https://github.com/BDomzal/magnetstein_data.git. MANIQ and ACD/TP project files are available at https://zenodo.org/ (doi: 10.5281/zenodo.8198722). The Python package with algorithm implementation is available at https://github.com/BDomzal/magnetstein. Code for reproducing experiments 1–11 is available at https://github.com/BDomzal/magnetstein_data.git.

## Supporting Information Available

Description of the spectra preprocessing, full results, library and mixture spectra for experiments 1–11, and main theoretical result with proof (PDF)
