# Supplementary material for: Magnetstein: An Open-Source Tool for Quantitative NMR Mixture Analysis Robust to Low Resolution, Distorted Lineshapes, and Peak Shifts
Source: Anal Chem. 2023 Dec 20;96(1):188–96. doi: 10.1021/acs.analchem.3c03594 (PMC10782418; doi:10.1021/acs.analchem.3c03594)
Supplement: Supplementary file 1 — ac3c03594_si_001.pdf [file ac3c03594_si_001.pdf]

Supporting Information

Magnetstein: An open source tool for quantitative NMR mixture analysis robust to low resolution, distorted lineshapes and peak shifts

Barbara Domżał[1], Ewa Klaudia Nawrocka[2], Dariusz Gołowicz[3], Michał Aleksander Ciach[1], Błażej Miasojedow[1], Krzysztof Kazimierczuk*[2], and Anna Gambin*[1]

[1]Faculty of Mathematics, Informatics and Mechanics, University of Warsaw, Banacha 2, Warsaw, 02-097, Poland.
[2]Centre of New Technologies, University of Warsaw, Banacha 2C, Warsaw, 02-097, Poland.
[3]Institute of Physical Chemistry, Polish Academy of Sciences, Kasprzaka 44/52, Warsaw, 01-224, Poland.

Contents

1 Preprocessing S2
2 Full results S3
3 The Wasserstein distance S16
4 Main theoretical result with proof S18

References **S31**

1 Preprocessing

In this section, we describe the standard preprocessing steps that we conduct before running estimation algorithm. The basic steps are the same for all the experiments. The procedure is as follows. First, we ensure that the data does not contain negative intensities or set them to zero. Next, we normalize both the components' spectra and mixture's spectrum. This is done by simply adding up all the intensities in the given spectrum and then dividing each intensity measurement in this spectrum by the obtained constant. After this operation, the sum of intenisties is equal to 1 for each spectrum. This step is necessary to perform Wasserstein-metric-based regression.

For experiments 2-5 and 8-11 we additionally cut spectra to regions of interest to avoid unnecessarily long computations. The remaining regions were: (0.53, 7.33) for Experiment 2, (-1.50, 10.50) for Experiment 3, (0.83, 4.13) for Experiments 4 and 9, (0.81, 4.11) for Experiment 5, (3.50, 12.00) for Experiment 8, (0.1, 4.0) for Experiments 10 and 11. Note that in Experiments 10-11 this step resulted in removing the peak of the solvent.

Steps described above is what we call 'basic' preprocessing in Table 1 in the main text.

Experiments 4, 5 and 9 (i.e. those involving metabolites) require some additional preprocessing steps. For those three experiments, we remove a peak from solvent present in mixture's spectrum. This is done simply by setting all the intenisties in region 4.4-4.8 to zeros. Moreover, in experiment 5, we shift the spectrum of a mixture by constant -0.14 in order to align it more with components' spectra. The data and the code, involving preprocessing, estimation and visualizations is fully available at `https://github.com/BDomzal/magnetstein_data.git`.

Table S 1: Results of estimation for all the experiments (some of them are not mentioned in the main text) obtained from Magnetstein with optimized parameters, Magnetstein with default parameters, ACD/TP and MANIQ.

Exp. no.	Problem	Ingredients	Ingredients' proportions					Settings	
			Real	Magnetstein optimized	Magnetstein default	ACD/TP	MANIQ	Preprocessing	Optimal parameters in Magnetstein
1	large intensity differences between peaks	α-pinene<br>benzyl benzoate<br>(in $CDCl_3$)	0.09<br>0.91	0.09<br>0.91	0.09<br>0.91	0.06<br>0.94	0.11<br>0.89	basic	$\kappa_{mixture} = 0.09$,<br>$\kappa_{components} = 0.08$
2	peak overlap<br>& contamination (extra peaks)	limonene<br>α-pinene<br>(in $CDCl_3$)	0.50<br>0.51	0.50<br>0.50	0.48<br>0.52	0.46<br>0.54	0.49<br>0.51	basic	$\kappa_{mixture} = 0.05$,<br>$\kappa_{components} = 0.01$
3	low resolution (43 MHz benchtop NMR spectrometer)	benzyl benzoate<br>isopropyl myristate<br>limonene<br>pinene	0.11<br>0.73<br>0.09<br>0.08	0.11<br>0.75<br>0.06<br>0.08	0.11<br>0.75<br>0.06<br>0.08	0.11<br>0.71<br>0<br>0.18	0.11<br>0.76<br>0.10<br>0.00	basic	$\kappa_{mixture} = 0.37$,<br>$\kappa_{components} = 0.24$
4	lineshape distortion<br>(shim z1 & z2)	lactate<br>alanine<br>creatine<br>creatinine<br>choline chloride<br>(in D_2O)	0.30<br>0.22<br>0.13<br>0.20<br>0.15	0.32<br>0.21<br>0.13<br>0.20<br>0.14	0.32<br>0.21<br>0.13<br>0.20<br>0.14	0.35<br>0.28<br>0.19<br>0.01<br>0.17	0.33<br>0.27<br>0<br>0.27<br>0.16	basic + removing peak from solvent (region 4.4-4.8)	$\kappa_{mixture} = 0.11$,<br>$\kappa_{components} = 0.02$
5	peak position mismatch between library and mixture (different temperatures)	lactate<br>alanine<br>creatine<br>creatinine<br>choline chloride<br>(in D_2O)	0.30<br>0.22<br>0.13<br>0.20<br>0.15	0.31<br>0.22<br>0.13<br>0.20<br>0.14	0.32<br>0.21<br>0.13<br>0.20<br>0.14	0.31<br>0.24<br>0.00<br>0.32<br>0.14	0.28<br>0.22<br>0.14<br>0.23<br>0.14	basic + removing peak from solvent (region 4.4-4.8) + shifting mixture's spectrum by constant -0.14	$\kappa_{mixture} = 0.03$,<br>$\kappa_{components} = 0.09$
6	test sample	α-pinene<br>benzyl benzoate<br>(in $CDCl_3$)	0.39<br>0.61	0.39<br>0.61	0.39<br>0.61	0.35<br>0.65	0.37<br>0.62	basic	$\kappa_{mixture} = 0.06$,<br>$\kappa_{components} = 0.2$
7	large intensity differences<br>& peak overlap	benzyl benzoate<br>m-anisaldehyde<br>(in $CDCl_3$)	0.84<br>0.16	0.84<br>0.16	0.84<br>0.16	0.83<br>0.17	0.85<br>0.15	basic	$\kappa_{mixture} = 0.23$,<br>$\kappa_{components} = 0.02$
8	peak overlap & different solvents for single ingredients (in $CDCl_3$) and mixture (DMSO-d_6)	benzyl benzoate<br>m-anisaldehyde	0.37<br>0.63	0.37<br>0.63	0.38<br>0.62	0.50<br>0.50	no result<br>no result	basic	$\kappa_{mixture} = 0.2$,<br>$\kappa_{components} = 0.29$
9	lineshape distortion (shim z2)	lactate<br>alanine<br>creatine<br>creatinine<br>choline chloride<br>(in D_2O)	0.30<br>0.22<br>0.13<br>0.20<br>0.15	0.32<br>0.21<br>0.13<br>0.20<br>0.14	0.32<br>0.21<br>0.13<br>0.20<br>0.14	0.43<br>0.18<br>0.19<br>0.01<br>0.18	0.28<br>0.22<br>0.14<br>0.23<br>0.14	basic + removing peak from solvent (region 4.4-4.8)	$\kappa_{mixture} = 0.05$,<br>$\kappa_{components} = 0.02$

2 Full results

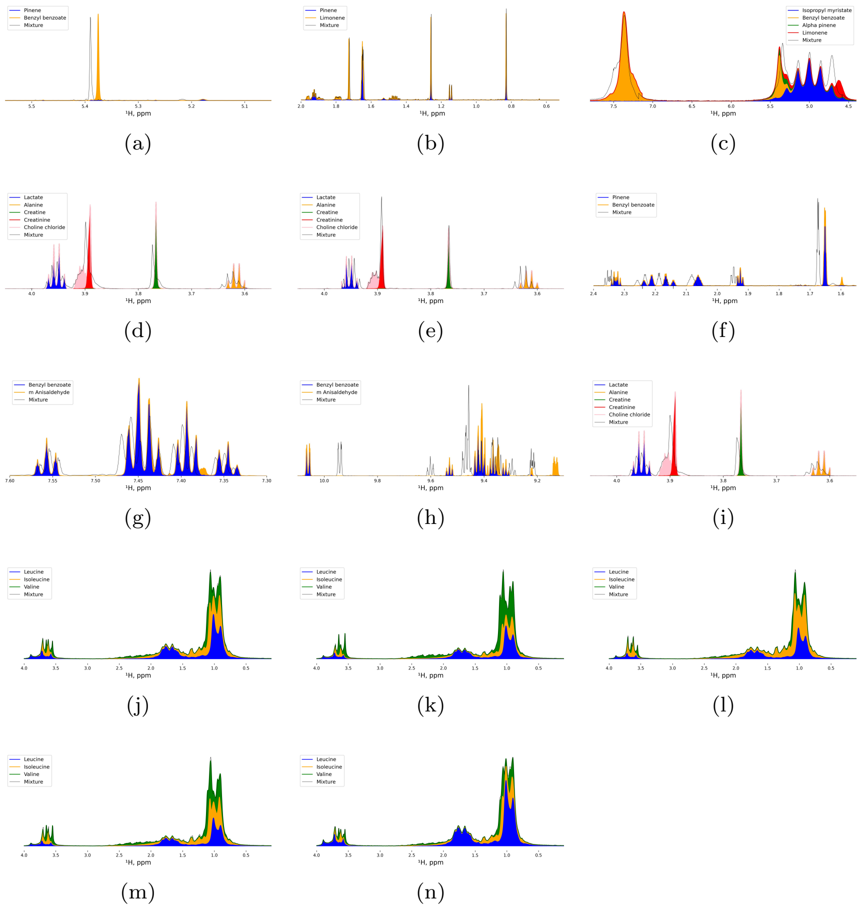

Figure S 1: Comparison of components' spectra added in proportions estimated by Magnetstein and mixture's spectrum for experiments 1-9, 10.1-10.4 and 11 (Supplementary Fig. 1a – 1n). For the sake of visibility, only parts of the spectra are shown.

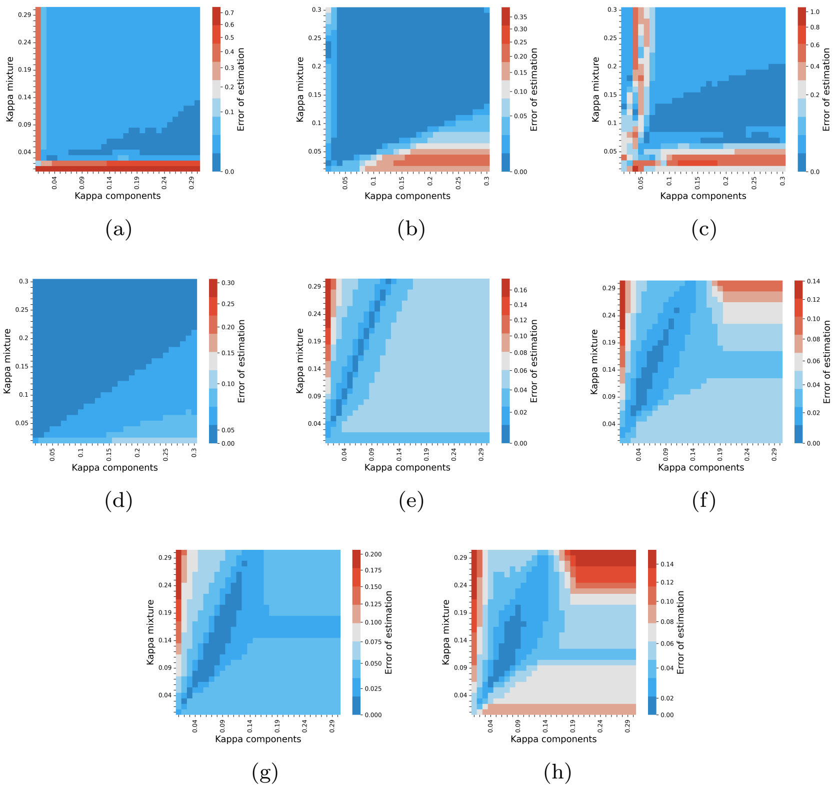

Figure S 2: Error of estimation for different values of κ_{mixture} and $\kappa_{\text{components}}$ parameters for Experiment 6 (Supplementary Fig. 2a), 7 (Supplementary Fig. 2b), 8 (Supplementary Fig. 2c), 9 (Supplementary Fig. 2d) and 10.1-10.4 (Supplementary Fig. 2e-2h). The error is defined as sum of absolute differences between the true value of proportion and the estimated value of proportion over all the components. For the default values of parameters (i.e. $\kappa_{\text{mixture}} = 0.25$, $\kappa_{\text{components}} = 0.22$) the proportion of noise in spectrum of a mixture is estimated as 0.0027, 0.0328, 0.0673, 0.0000, 0.0247, 0.0202, 0.0201 and 0.0153 for experiments 6-9, 10.1-10.4, respectively. The proportion of noise in linear combination of components is estimated as 0.0095, 0.0223, 0.0107, 0.0130, 0.0000, 0.0000, 0.0000 and 0.0000 for experiments 6-9, 10.1-10.4, respectively.

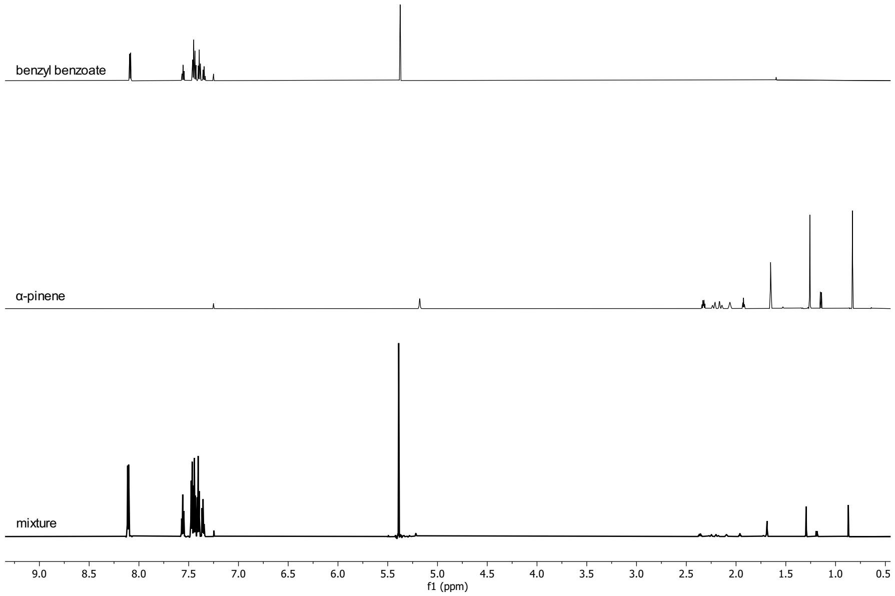

Figure S 3: Spectra of library components and mixture from the Experiment no. 1. For details see Table S1.

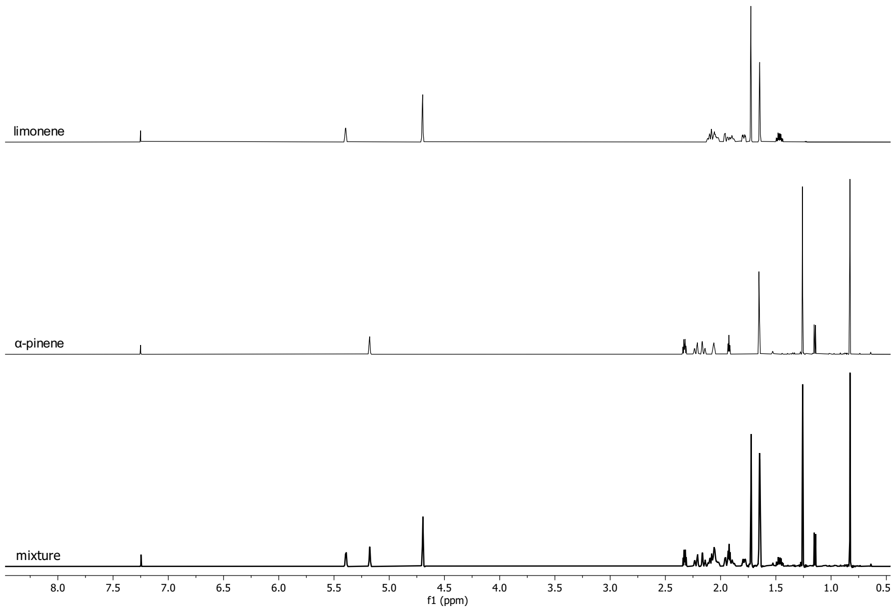

Figure S 4: Spectra of library components and mixture from the Experiment no. 2. For details see Table S1.

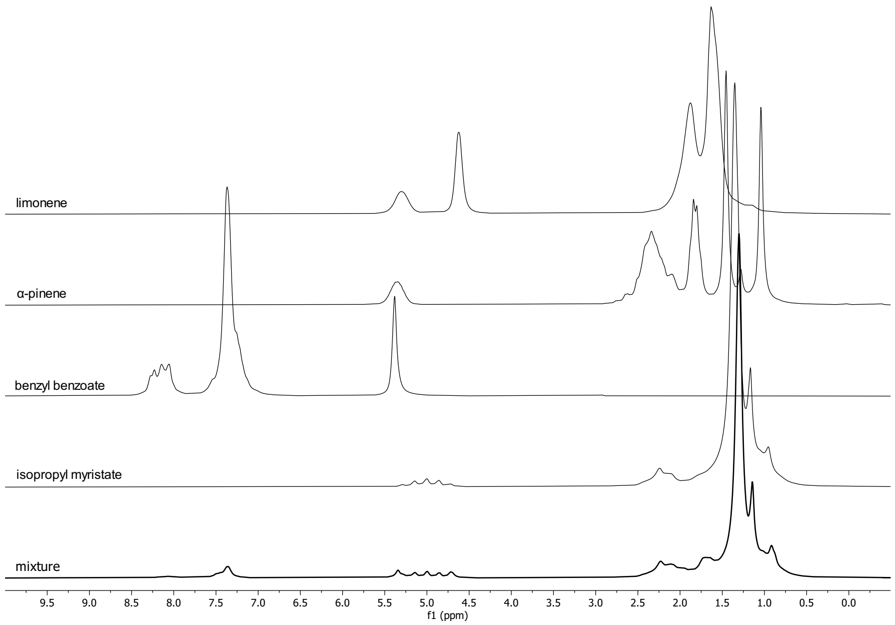

Figure S 5: Spectra of library components and mixture from the Experiment no. 3. For details see Table 1.

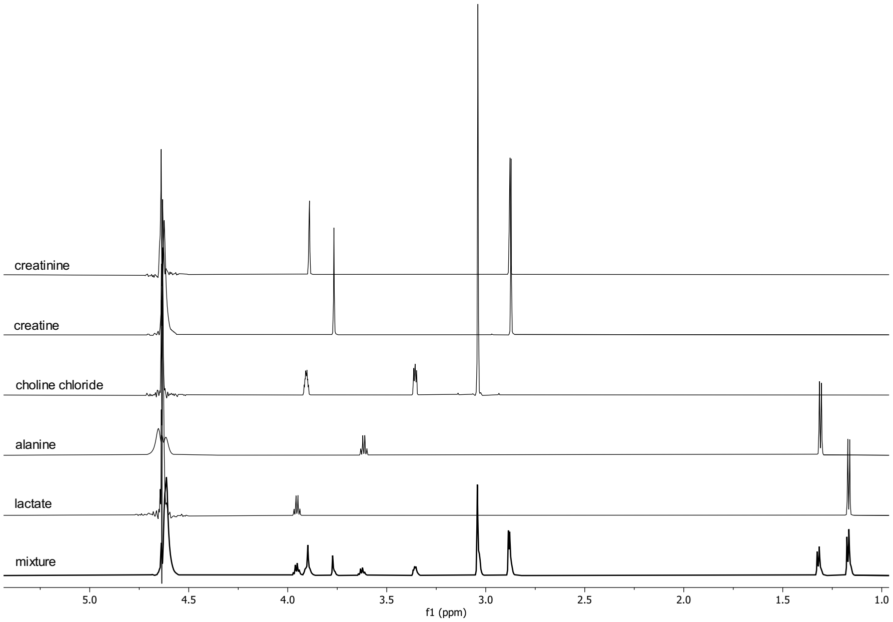

Figure S 6: Spectra of library components and mixture from the Experiment no. 4. For details see Table S1.

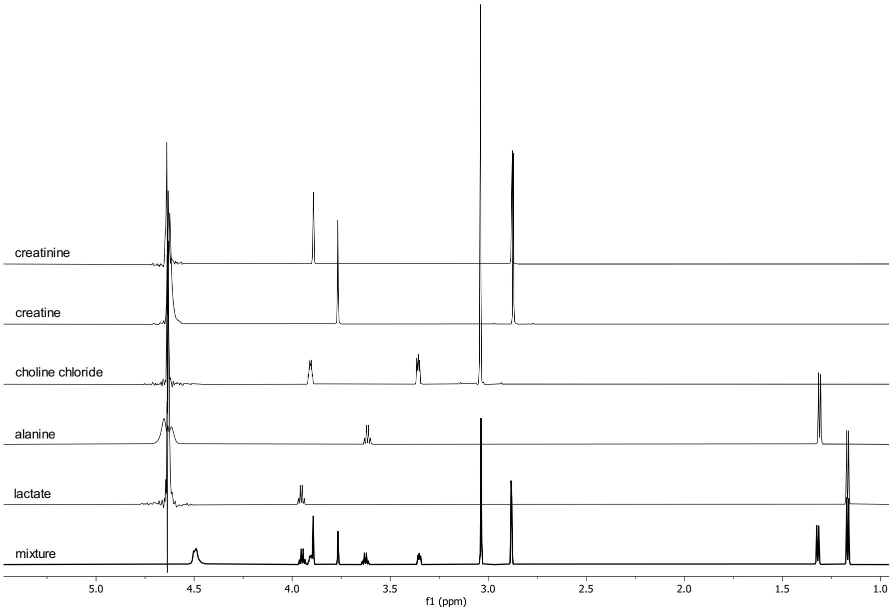

Figure S 7: Spectra of library components and mixture from the Experiment no. 5. For details see Table S1.

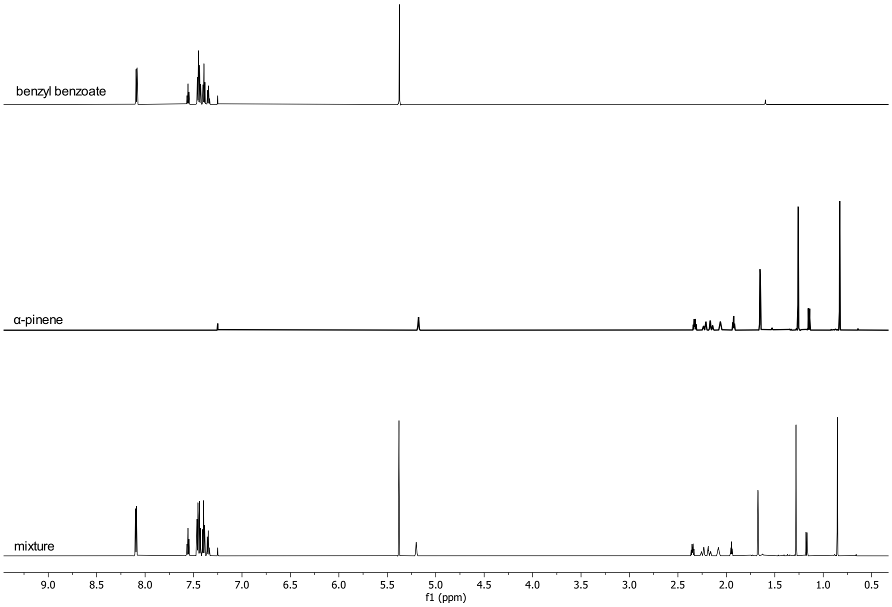

Figure S 8: Spectra of library components and mixture from the Experiment no. 6. For details see Table S1.

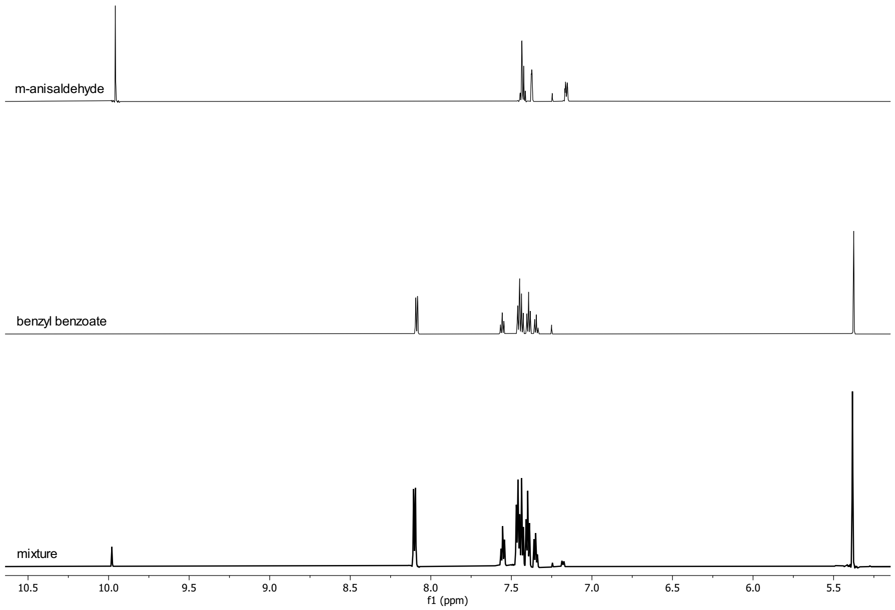

Figure S 9: Spectra of library components and mixture from the Experiment no. 7. For details see Table S1.

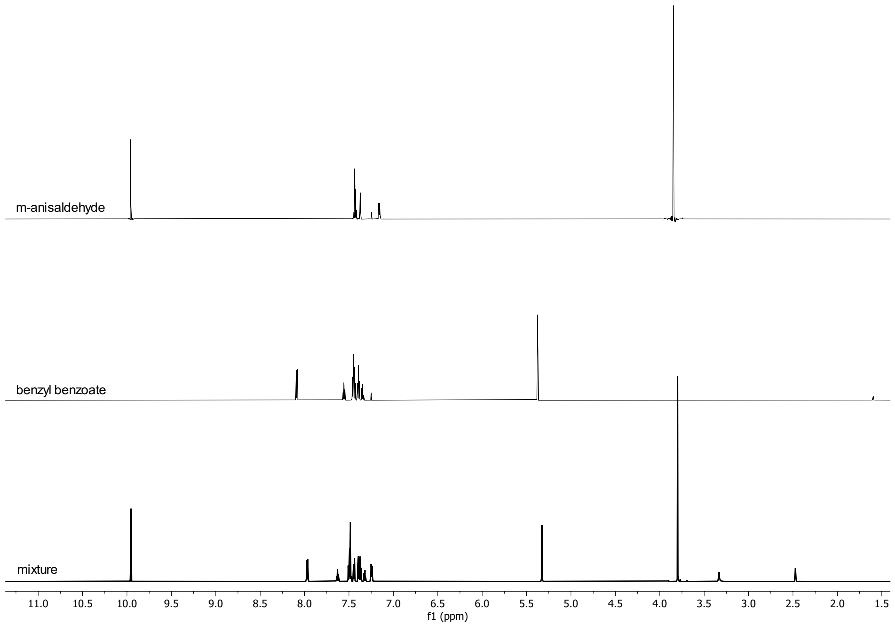

Figure S 10: Spectra of library components and mixture from the Experiment no. 8. For details see Table S1.

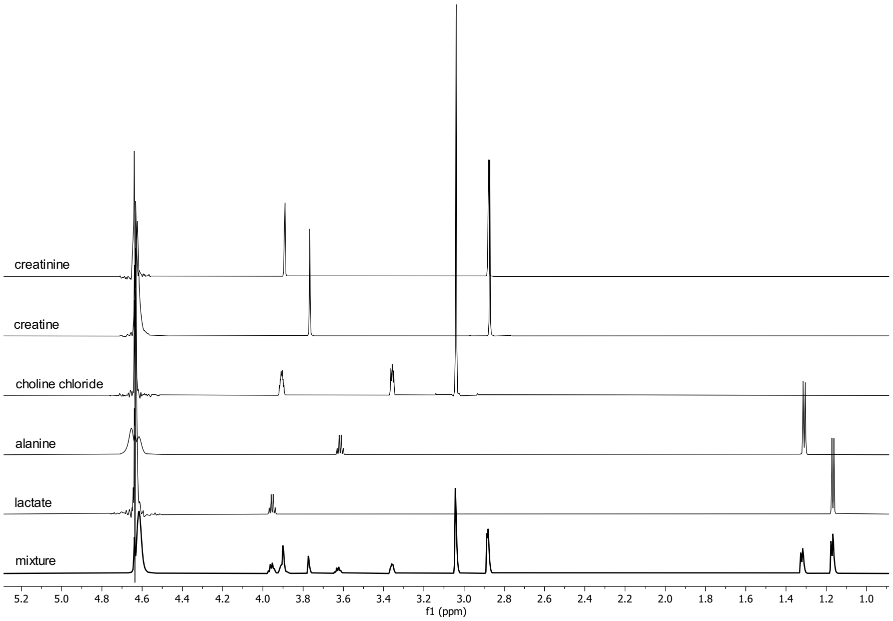

Figure S 11: Spectra of library components and mixture from the Experiment no. 9. For details see Table S1.

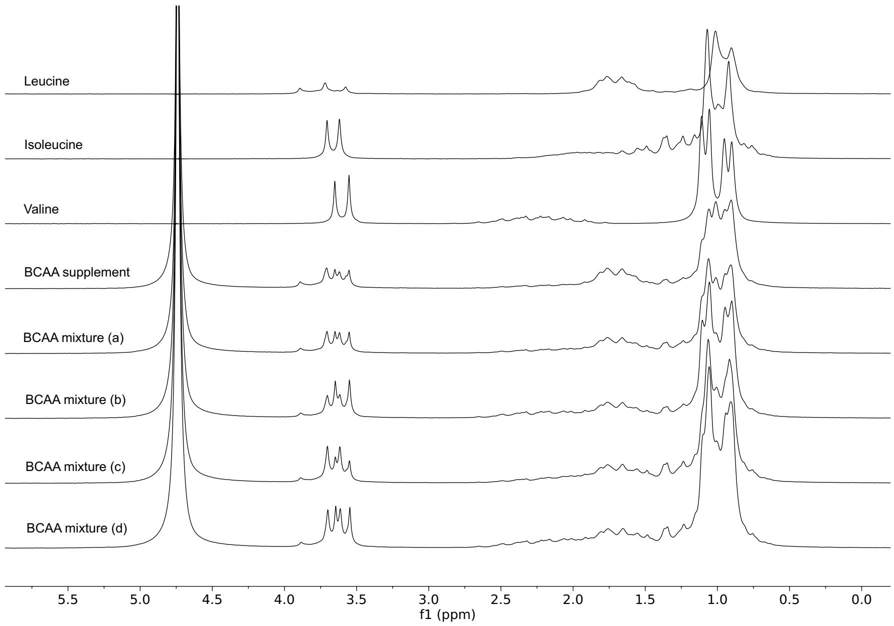

Figure S 12: Spectra of library components and mixtures from Experiments 10 and 11. For details, see the Experimental Section in the main text.

3 The Wasserstein distance

To introduce the concept of the Wasserstein distance for spectra comparison, we first need to represent a spectrum as a probability measure. This approach is somewhat different than the standard vector representation. The main difference between the two approaches is that the vector representation implicitly assumes the chemical shift positions for consecutive intensity values and thus makes it impossible to compare or add two spectra with different points on chemical shift axis. On the contrary, probability measure respresentation does not require common chemical shift axis for all the spectra, because it encodes the spectrum explicitly as a set of tuples. The difference is visualized in Figure 13.

Below, we formally introduce the notion of the Wasserstein distance. We only describe one-dimensional case here, as it is sufficient for the purposes of this paper.

Definition 1. *Let $X \subset \mathbb{R}$ and let $\mathcal{P}(X)$ be the space of probability measures defined on X. Let's define $\mathcal{P}_1(X)$ as:*

$$\mathcal{P}_1(X) := \{\mu \in \mathcal{P}(X) : \int_X |x| d\mu(x) < +\infty\}.$$

Definition 2. *Let $\mu \in \mathcal{P}_1(X)$, $\nu \in \mathcal{P}_1(Y)$. A probability measure $\gamma : X \times Y \to [0,1]$ satisfying conditions:*

$$\int_{y \in Y} d\gamma(x,y) = \mu(x), \qquad \int_{x \in X} d\gamma(x,y) = \nu(y)$$

is called a transport plan between μ and ν. We denote a set of all transport plans between μ and ν with $\Gamma = \Gamma(\mu,\nu)$.

Definition 3. *Let μ, $\nu \in \mathcal{P}_1(X)$. We define the Wasserstein distance (the Wasserstein metric) between μ and ν as*

$$W_{|.|}(\mu,\nu) = \min_{\gamma \in \Gamma(\mu,\nu)} \int_{X \times X} |x-y| d\gamma(x,y).$$

In a more general formulation, absolute value in the above definition can be replaced by some other distance function $\rho : X \times X \to [0, +\infty)$. Then we have:

$$W_{\rho}(\mu,\nu) = \min_{\gamma \in \Gamma(\mu,\nu)} \int_{X \times X} \rho(x,y) d\gamma(x,y).$$

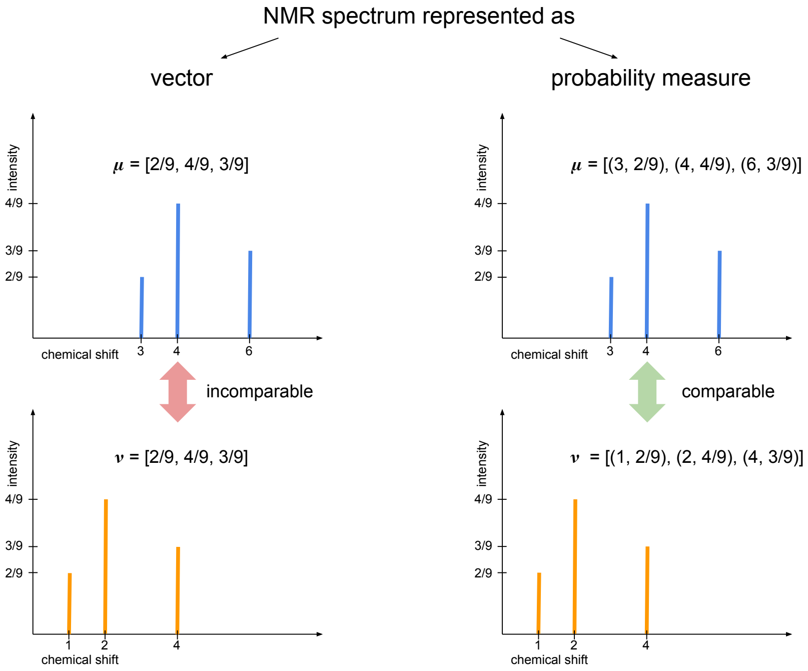

Figure S 13: Illustration of two approaches to representing spectra: as a vector (left) and as a probability measure (right).

4 Main theoretical result with proof

The main goal of estimation is finding p^* such that

$$p^* = \underset{p=(p'_0, p_0, p_1, \ldots, p_k)}{\arg\min} W_{\rho_2}((1-p'_0)\mu + p'_0\xi, \nu_p + p_0\omega), \tag{1}$$

where μ is spectrum of a mixture, $\nu_p = \sum_{j=1}^{k} p_j \nu_j$ is a linear combination of reference spectra $\nu_1, \ldots, \nu_k$ and ω, ξ are auxiliary points corresponding to noise in the spectrum of a mixture and reference spectra of components, respectively. For all $p = (p'_0, p_0, p_1, \ldots, p_k)$ we have

$$\begin{aligned} p_0 + \sum_{i=1}^{k} p_i &= 1, \\ 0 \leq p'_0, p_0, p_1, \ldots, p_k &\leq 1. \end{aligned}$$

Moreover, we assume that the mixture's spectrum and the reference spectra are normalized, i.e.:

$$\begin{aligned} \int_{x \in \mathbb{R}} d\mu(x) &= 1, \\ \int_{y \in \mathbb{R}} d\nu_j(y) &= 1 \text{ for } j = 1, 2, \ldots, k. \end{aligned}$$

Additionally, we need to assume that

$$1 - p_0 - p'_0 \geq 0. \tag{2}$$

This assumption guarantees that the amount of signal removed to ω is not greater than the amount of signal present in rescaled mixture's spectrum (i.e. $1 - p'_0 \geq p_0$) and the amount of signal removed to ξ is not greater than the amount of signal present in reference spectra (i.e. $1 - p_0 \geq p'_0$).

For simplicity of notation, let us denote κ_{mixture} (penalty for removing noise signal from spectrum μ) as κ and $\kappa_{\text{components}}$ (penalty for removing signal from spectrum ν_p) as κ'.

Below we present our main theoretical result:

Theorem 1. *Let $S = \{s_1, s_2, \ldots, s_n\}$ be an ordered list of all distinct ppm values present in all the spectra. Problem of finding*

$$p^* = \arg\min_p W_{\rho_2}((1-p'_0)\mu + p'_0\xi, \nu_p + p_0\omega)$$

with distance function

$$\rho_2(x, y) = \begin{cases} |x - y| & \text{if } x, y \in \mathbb{R}, \\ \kappa & \text{if } x \in \mathbb{R}, y = \omega, \\ \kappa' & \text{if } x = \xi, y \in \mathbb{R}, \\ \kappa + \kappa' & \text{if } x = \xi, y = \omega. \end{cases}$$

is equivalent to a linear program

$$\begin{aligned}
\text{maximize} \quad & V^T \boldsymbol{z} \\
\text{subject to} \quad & (V')^T \boldsymbol{z} \leq \kappa', \\
& W^T \boldsymbol{z} \leq -\kappa, \\
& z_i - z_n \leq 0 \quad \textit{for } i = 1, 2, ..., n-1, \\
& -z_i - z_{n+1} \leq 0 \quad \textit{for } i = 1, 2, ..., n-1, \\
& z_i - z_{i+1} \leq l_i \quad \textit{for } i = 1, 2, ..., n-2, \\
& z_{n-1} \leq l_{n-1}, \\
& z_i - z_{i+1} \geq -l_i \quad \textit{for } i = 1, 2, ..., n-2, \\
& z_{n-1} \geq -l_{n-1}, \\
& z_n, z_{n+1}, z_{n+2}, z_{n+3} \geq 0, \\
& \boldsymbol{z} \in \mathbb{R}^{n+3},
\end{aligned}$$

where

$$V := \begin{bmatrix} \mu(s_1) & \mu(s_2) & ... & \mu(s_{n-1}) & -1 & 0 & 0 & -1 \end{bmatrix}^T,$$

$$V' := \begin{bmatrix} \mu(s_1) & \mu(s_2) & ... & \mu(s_{n-1}) & 0 & 1 & -1 & 0 \end{bmatrix}^T,$$

$$W := \begin{bmatrix} \nu_1(s_1) & \nu_2(s_1) & ... & \nu_k(s_1) \\ \nu_1(s_2) & \nu_2(s_2) & ... & \nu_k(s_2) \\ & & ... & \\ \nu_1(s_{n-1}) & \nu_2(s_{n-1}) & ... & \nu_k(s_{n-1}) \\ -1 & -1 & ... & -1 \\ 0 & 0 & ... & 0 \\ 1 & 1 & ... & 1 \\ -1 & -1 & ... & -1 \end{bmatrix},$$

$$l_i := s_{i+1} - s_i.$$

Let's denote by $M(s)$ and $N(s) = \sum_{j=1}^{k} p_j N_j(s)$ cumulative distribution functions of μ and ν_p, respectively. We are going to prove our main theoretical result, but first, let's recall a useful theorem proved in [2]:

Theorem 2. *Let μ and ν be two probability measures on the real line $\mathbb{R}$. Let M and N be the cumulative distribution function of μ and ν respectively. Then*

$$W_{|.|}(\mu, \nu) = \int_{\mathbb{R}} |M(s) - N(s)| ds.$$

Now, we are going to prove the theorem on equivalence by splitting it into Theorem 3 and Theorem 4. Theorem 4 provides computationally convenient way of finding optimal proportions.

Theorem 3. *Let $\gamma \in \Gamma$ be a transport plan, $g(x) = \gamma(x,\omega)$, $g'(y) = \gamma(\xi, y)$ and let $G(s)$ and $G'(s)$ be the cumulative distribution functions of g and g', respectively. Problem of finding*

$$p^* = \arg\min_{p} W_{\rho_2}((1-p_0')\mu + p_0'\xi, \nu_p + p_0\omega)$$

with distance function

$$\rho_2(x,y) = \begin{cases} |x-y| & \text{if } x,y \in \mathbb{R}, \\ \kappa & \text{if } x \in \mathbb{R}, y=\omega, \\ \kappa' & \text{if } x=\xi, y \in \mathbb{R}, \\ \kappa+\kappa' & \text{if } x=\xi, y=\omega. \end{cases}$$

and problem

$$p^*, g^*, g'^* = \arg\min_{p,g,g'} \left\{ \kappa p_0 + \kappa' p_0' + \sum_{i=1}^{n-1} (s_{i+1}-s_i) \Big| (1-p_0')M(s_i) + G'(s_i) - G(s_i) - N_p(s_i) \Big| \right\}$$

are equivalent.

Proof. Using the definition of the distance function ρ_2, we can reformulate the Wasserstein distance between $(1-p_0')\mu + p_0'\xi$ and $\nu_p + p_0\omega$ as:

$$\begin{aligned}
W_{\rho_2} \quad & ((1-p_0')\mu + p_0'\xi, p_0\omega + p_1\nu_1 + \ldots + p_k\nu_k) = \\
= \quad & \min_{\gamma\in\Gamma} \int_{x\in\mathbb{R}\cup\{\xi\}} \int_{y\in\mathbb{R}\cup\{\omega\}} \rho_2(x,y) d\gamma(x,y) \\
= \quad & \min_{\gamma\in\Gamma} \Big(\int_{x\in\mathbb{R}} \int_{y\in\mathbb{R}} |x-y| d\gamma(x,y) + \int_{x\in\mathbb{R}} \kappa d\gamma(x,\omega) + \int_{y\in\mathbb{R}} \kappa' d\gamma(\xi,y) + (\kappa+\kappa')\gamma(\xi,\omega) \Big) \\
= \quad & \min_{\gamma\in\Gamma} \Big(\int_{x\in\mathbb{R}} \int_{y\in\mathbb{R}} |x-y| d\gamma(x,y) + \kappa(p_0 - \gamma(\xi,\omega)) + \kappa'(p_0' - \gamma(\xi,\omega)) + (\kappa+\kappa')\gamma(\xi,\omega) \Big) \\
= \quad & \min_{\gamma\in\Gamma} \Big(\kappa p_0 + \kappa' p_0' + \int_{x\in\mathbb{R}} \int_{y\in\mathbb{R}} |x-y| \gamma(x,y) dy dx \Big) \\
= \quad & \kappa p_0 + \kappa' p_0' + \min_{\gamma\in\Gamma} \int_{x\in\mathbb{R}} \int_{y\in\mathbb{R}} |x-y| \gamma(x,y) dy dx
\end{aligned}$$

To use Theorem 2, we need to have a probability measure defined on $\mathbb{R}^2$, but this is not the case yet. Let's recall that $g(x) = \gamma(x,\omega)$, $g'(y) = \gamma(\xi,y)$ and $G(s)$ and $G'(s)$ are cumulative distribution functions of g and g', respectively. Note that for all $x \in \mathbb{R}$

$$\int_{y\in\mathbb{R}} \gamma(x,y) dy \quad = \quad (1-p_0')\mu(x) - g(x), \tag{3}$$

$$\int_{x\in\mathbb{R}} dg(x) \quad = \quad p_0 - \gamma(\xi,\omega). \tag{4}$$

Moreover, from assumption $1-p_0-p_0' \geq 0$, we infer that

$$1-p_0-p_0'+\gamma(\xi,\omega) \geq 0. \tag{5}$$

Now, we are going to show that $\gamma_{|\mathbb{R}^2}(x,y)/(1-p_0-p_0'+\gamma(\xi,\omega))$ is a probability measure. Indeed, we have:

$$\begin{aligned}\int_{x\in\mathbb{R}}\int_{y\in\mathbb{R}}\gamma_{|\mathbb{R}^2}(x,y)/(1-p_0-p_0'+\gamma(\xi,\omega))dydx &= \frac{\int_{x\in\mathbb{R}}((1-p_0')\mu(x)-g(x))dx}{1-p_0-p_0'+\gamma(\xi,\omega)}\\ &= \frac{1-p_0'-p_0+\gamma(\xi,\omega)}{1-p_0-p_0'+\gamma(\xi,\omega)}\\ &= 1,\end{aligned}$$

where the first equality follows from (3) and the second equality follows from (4) and the fact that μ is a probability measure. We can also find marginal distributions of $\gamma_{|\mathbb{R}^2}(x,y)/(1-p_0-p_0'+\gamma(\xi,\omega))$ as we are going to need them in the subsequent part of the proof:

$$\int_{y\in\mathbb{R}}\frac{\gamma_{|\mathbb{R}^2}(x,y)}{(1-p_0-p_0'+\gamma(\xi,\omega))}dy = \frac{(1-p_0')\mu(x)-g(x)}{1-p_0-p_0'+\gamma(\xi,\omega)}, \tag{6}$$

$$\int_{x\in\mathbb{R}}\frac{\gamma_{|\mathbb{R}^2}(x,y)}{(1-p_0-p_0'+\gamma(\xi,\omega))}dx = \frac{p_1\nu_1(y)+...+p_k\nu_k(y)-g'(y)}{1-p_0-p_0'+\gamma(\xi,\omega)}. \tag{7}$$

Knowing that $M(s), N(s), G(s)$ and $G'(s)$ are cumulative distribution functions of μ, ν_p, g and g' respectively, we can compute cumulative distribution functions of the marginals (6)–(7):

$$\int_{-\infty}^{s}\frac{(1-p_0')\mu(x)-g(x)}{1-p_0-p_0'+\gamma(\xi,\omega)}dx = \frac{(1-p_0')M(s)-G(s)}{1-p_0-p_0'+\gamma(\xi,\omega)}, \tag{8}$$

$$\int_{-\infty}^{s}\frac{\nu_p(y)-g'(y)}{1-p_0-p_0'+\gamma(\xi,\omega)}dy = \frac{N(s)-G'(s)}{1-p_0-p_0'+\gamma(\xi,\omega)} \tag{9}$$

Now, we can apply Theorem 2 to our problem. We split minimization over all possible transport plans γ into minimization over $\gamma_{|\mathbb{R}^2}$ and over $g, g', \gamma(\xi,\omega)$:

$$\begin{aligned}\min_{\gamma\in\Gamma}\quad & \int_{x\in\mathbb{R}}\int_{y\in\mathbb{R}}|x-y|\gamma(x,y)dydx\\ &= \min_{g,g',\gamma(\xi,\omega)}\min_{\gamma_{|\mathbb{R}^2}}\int_{x\in\mathbb{R}}\int_{y\in\mathbb{R}}|x-y|\gamma(x,y)dydx\\ &= \min_{g,g',\gamma(\xi,\omega)}(1-p_0-p_0'+\gamma(\xi,\omega))\min_{\gamma_{|\mathbb{R}^2}}\int_{x\in\mathbb{R}}\int_{y\in\mathbb{R}}\frac{|x-y|\gamma(x,y)}{1-p_0-p_0'+\gamma(\xi,\omega)}dydx\\ &= \min_{g,g',\gamma(\xi,\omega)}(1-p_0-p_0'+\gamma(\xi,\omega))\int_{s\in\mathbb{R}}\left|\frac{(1-p_0')M(s)-G(s)}{1-p_0-p_0'+\gamma(\xi,\omega)}-\frac{N(s)-G'(s)}{1-p_0-p_0'+\gamma(\xi,\omega)}\right|ds\\ &= \min_{g,g',\gamma(\xi,\omega)}\int_{s\in\mathbb{R}}\left|(1-p_0')M(s)-G(s)-N(s)+G'(s)\right|ds,\\ &= \min_{g,g'}\int_{s\in\mathbb{R}}\left|(1-p_0')M(s)-G(s)-N(s)+G'(s)\right|ds,\end{aligned}$$

where the third equality is a consequence of Theorem 2, the fact that $\gamma_{|\mathbb{R}^2}(x,y)/(1-p_0-p'_0+\gamma(\xi,\omega))$ is a probability measure and equations (8)–(9). In fourth equality we use the fact (5). The last equality holds, because the minimized expression does not depend on the choice of $\gamma(\xi,\omega)$.

Finally, we obtain:

$$W_{\rho_2}((1-p'_0)\mu+p'_0\xi, p_0\omega+p_1\nu_1+...+p_k\nu_k) \\ = \kappa p_0+\kappa' p'_0+\min_{g,g'} \int_{s\in\mathbb{R}} \Big|(1-p'_0)M(s)-G(s)-N(s)+G'(s)\Big|ds.$$

Term $\kappa p_0+\kappa' p'_0$ does not depend on g nor g', so we get:

$$\min_p W_{\rho_2}((1-p'_0)\mu+p'_0\xi, p_0\omega+p_1\nu_1+...+p_k\nu_k) \\ = \min_{p,g,g'} \Big(\kappa p_0+\kappa' p'_0+\int_{s\in\mathbb{R}} \Big|(1-p'_0)M(s)-G(s)-N(s)+G'(s)\Big|ds\Big).$$

In the discrete case that is exactly our thesis. □

So, as we see from Theorem 3, instead of the original formulation we can now consider:

$$p^*, g^*, g'^* = \arg\min_{p,g,g'} \Big\{\kappa_0 p_0+\kappa' p'_0+\sum_{i=1}^{n-1}(s_{i+1}-s_i)\Big|(1-p'_0)M(s_i)+G'(s_i)-G(s_i)-N_p(s_i)\Big|\Big\}. \tag{10}$$

To solve this problem, we use linear programming.

Theorem 4. *Problem*

$$p^*, g^*, g'^* = \arg\min_{p,g,g'} \Big\{\kappa p_0+\kappa' p'_0+\sum_{i=1}^{n-1}(s_{i+1}-s_i)\Big|(1-p'_0)M(s_i)+G'(s_i)-G(s_i)-N_p(s_i)\Big|\Big\}$$

is equivalent to

$$\begin{aligned} \text{maximize} \quad & V^T\boldsymbol{z} \\ \text{subject to} \quad & (V')^T\boldsymbol{z} \le \kappa', \\ & W^T\boldsymbol{z} \le -\kappa, \\ & z_i - z_n \le 0 \quad \textit{for } i=1,2,...,n-1, \\ & -z_i - z_{n+1} \le 0 \quad \textit{for } i=1,2,...,n-1, \\ & z_i - z_{i+1} \le l_i \quad \textit{for } i=1,2,...,n-2, \\ & z_{n-1} \le l_{n-1}, \\ & z_i - z_{i+1} \ge -l_i \quad \textit{for } i=1,2,...,n-2, \\ & z_{n-1} \ge -l_{n-1}, \\ & z_n, z_{n+1}, z_{n+2}, z_{n+3} \ge 0, \\ & \boldsymbol{z} \in \mathbb{R}^{n+3}, \end{aligned}$$

where

$$V := \begin{bmatrix}\mu(s_1) & \mu(s_2) & ... & \mu(s_{n-1}) & -1 & 0 & 0 & -1\end{bmatrix}^T,$$

$$V' := \begin{bmatrix}\mu(s_1) & \mu(s_2) & ... & \mu(s_{n-1}) & 0 & 1 & -1 & 0\end{bmatrix}^T,$$

$$W := \begin{bmatrix} \nu_1(s_1) & \nu_2(s_1) & ... & \nu_k(s_1) \\ \nu_1(s_2) & \nu_2(s_2) & ... & \nu_k(s_2) \\ & & ... & \\ \nu_1(s_{n-1}) & \nu_2(s_{n-1}) & ... & \nu_k(s_{n-1}) \\ -1 & -1 & ... & -1 \\ 0 & 0 & ... & 0 \\ 1 & 1 & ... & 1 \\ -1 & -1 & ... & -1 \end{bmatrix},$$

$$l_i := s_{i+1} - s_i$$

and $S = \{s_1, s_2, ..., s_n\}$ *is an ordered list of all distinct ppm values present in all the spectra.*

Proof. Let's denote:

$$\begin{aligned} l_i &:= s_{i+1} - s_i, \\ M_i &:= M(s_i), \\ G'_i &:= G'(s_i), \\ G_i &:= G(s_i), \\ N_{ij} &:= N_j(s_i), \\ g'_i &:= g'(s_i), \\ g_i &:= g(s_i), \\ \epsilon_i &:= (1-p_0)M_i + G'_i - G_i - \sum_{j=1}^{k} N_{ij}p_j. \end{aligned}$$

Using this notation, we can rewrite our minimization problem as:

$$\begin{aligned}
\text{minimize} \quad & \sum_{i=1}^{n-1} l_i|\epsilon_i| + \kappa p_0 + \kappa' p'_0 \quad \text{over } \epsilon, p, g, g' \\
\text{subject to} \quad & \epsilon_i + \sum_{j=1}^{i} g_j - \sum_{j=1}^{i} g'_j + \sum_{j=1}^{k} N_{ij} p_j + p'_0 M_i = M_i \quad \text{for } i = 1, 2, ..., n-1, & (11) \\
& \sum_{i=1}^{n} g_i \leq p_0, & (12) \\
& \sum_{i=1}^{n} g'_i \leq p'_0, & (13) \\
& 1 - p_0 - p'_0 \geq 0, & (14) \\
& \sum_{j=1}^{k} p_j \leq 1, & (15) \\
& \epsilon_i^+, \epsilon_i^-, p'_0, p_j, g_i, g'_i \geq 0.
\end{aligned}$$

Constraint (11) is a consequence of definition of ϵ_i, (12) and (13) follow from the fact that the total amount of signal removed from experimental and theoretical spectra cannot be greater than proportions p_0 and p'_0, respectively. We do not have equality here, because we leave open the possibility that some amount of signal, $\gamma(\xi, \omega)$, will be transported from ξ to ω. However, this situation lacks clear interpretation and, as we show later, is never optimal. Therefore, it will not happen in practice. Constraint (14) guarantees that the amount of signal removed to ω is not greater than the amount of signal present in rescaled experimental spectrum (i.e. $1 - p'_0 \geq p_0$) and the amount of signal removed to ξ is not greater than the amount of signal present in reference spectra (i.e. $1 - p_0 \geq p'_0$). Finally, (15) states that the sum of proportions must be less than 1.

To obtain a linear program in a standard form, we need to have linear function and non-negative variables. We split ϵ_i and $|\epsilon_i|$ using positive and negative parts:

$$\begin{aligned}
\epsilon_i &= \epsilon_i^+ - \epsilon_i^-, \\
|\epsilon_i| &= \epsilon_i^+ + \epsilon_i^-.
\end{aligned}$$

We can also get rid of p_0 by substituting

$$p_0 = 1 - \sum_{j=1}^{k} p_j$$

and then we obtain:

$$\begin{aligned}
&\text{minimize} && \sum_{i=1}^{n-1} l_i\epsilon_i^+ + \sum_{i=1}^{n-1} l_i\epsilon_i^- - \kappa\sum_{j=1}^{k} p_k + \kappa' p_0' \quad \text{over } \epsilon^+, \epsilon^-, p, g, g' \\
&\text{subject to} && \epsilon_i^+ - \epsilon_i^- + \sum_{j=1}^{i} g_j - \sum_{j=1}^{i} g_j' + \sum_{j=1}^{k} N_{ij}p_j + p_0' M_i = M_i \quad \text{for } i = 1, 2, ..., n-1, \\
& && -\sum_{i=1}^{n} g_i - \sum_{j=1}^{k} p_j \geq -1, \\
& && p_0' - \sum_{i=1}^{n} g_i' \geq 0, \\
& && \sum_{j=1}^{k} p_j - p_0' \geq 0, \\
& && -\sum_{j=1}^{k} p_j \geq -1, \\
& && \epsilon_i^+, \epsilon_i^-, p_0', p_j, g_i, g_i' \geq 0,
\end{aligned}$$

which is a linear program. (Note that we skip a constant $+\kappa$ in the objective function, since minimizing an expression plus constant is equivalent to minimizing an expression itself). Now, to simplify the computations, let's find its dual

formulation:

$$\begin{array}{ll}
\text{maximize} & \displaystyle\sum_{i=1}^{n-1} M_i x_i - x_n - x_{n+3} \\
\text{subject to} & x_i \leq l_i \quad \text{for } i = 1, ..., n-1, \\
& -x_i \leq l_i \quad \text{for } i = 1, ..., n-1, \\
& \displaystyle\sum_{k=i}^{n-1} x_k - x_n \leq 0 \quad \text{for } i = 1, ..., n-1, \\
& -x_n \leq 0, \\
& -\displaystyle\sum_{k=i}^{n-1} x_k - x_{n+1} \leq 0 \text{ for } i = 1, ..., n-1, \\
& -x_{n+1} \leq 0, \\
& \displaystyle\sum_{i=1}^{n-1} M_i x_i + x_{n+1} - x_{n+2} \leq \kappa', \\
& \displaystyle\sum_{i=1}^{n-1} N_{ij} x_i - x_n + x_{n+2} - x_{n+3} \leq -\kappa \text{ for } j = 1, ..., k, \\
& x_n, x_{n+1}, x_{n+2}, x_{n+3} \geq 0, \\
& \mathbf{x} \in \mathbb{R}^{n+3}.
\end{array}$$

In order to use matrix notation, let's define $L \in \mathbb{R}^{n-1}$, $M \in \mathbb{R}^{n-1}$, $U \in \mathbb{M}_{(n-1)\times(n-1)}$, $N \in \mathbb{M}_{(n-1)\times k}$, $\widetilde{V} \in \mathbb{R}^{n-1}$, $\widetilde{W} \in \mathbb{M}_{(n-1)\times k}$ as:

$$L := \begin{bmatrix} l_1 & l_2 & ... & l_{n-1} \end{bmatrix}^T,$$

$$M := \begin{bmatrix} M_1 & M_2 & ... & M_{n-1} \end{bmatrix}^T,$$

$$U := \begin{bmatrix} 1 & 0 & ... & 0 \\ 1 & 1 & ... & 0 \\ & & ... & \\ 1 & 1 & ... & 1 \end{bmatrix},$$

$$N := \begin{bmatrix} N_{11} & N_{12} & ... & N_{1k} \\ N_{21} & N_{22} & ... & N_{2k} \\ & & ... & \\ N_{(n-1)1} & N_{(n-1)2} & ... & N_{(n-1)k} \end{bmatrix},$$

$$\widetilde{V} := \begin{bmatrix} \mu(s_1) & \mu(s_2) & ... & \mu(s_{n-1}) \end{bmatrix}^T,$$

$$\widetilde{W} := \begin{bmatrix} \nu_1(s_1) & \nu_2(s_1) & ... & \nu_k(s_1) \\ \nu_1(s_2) & \nu_2(s_2) & ... & \nu_k(s_2) \\ & & ... & \\ \nu_1(s_{n-1}) & \nu_2(s_{n-1}) & ... & \nu_k(s_{n-1}) \end{bmatrix}.$$

Let's also split $\mathbf{x}$ into two vectors:

$$\begin{aligned}\widetilde{\mathbf{x}} &= \begin{bmatrix} x_1 & x_2 & ... & x_{n-1} \end{bmatrix}^T, \\ \widehat{\mathbf{x}} &= \begin{bmatrix} x_n & x_{n+1} & x_{n+2} & x_{n+3} \end{bmatrix}^T\end{aligned}$$

Note that $M = U\widetilde{V}$ and $N = U\widetilde{W}$. Using matrices $\widetilde{V}$ and $\widetilde{W}$ in computations is much more efficient than using M and N, as they are sparse. Now, we can rewrite the dual program as:

$$\text{maximize} \qquad \widetilde{V}^T U^T \widetilde{\mathbf{x}} + \begin{bmatrix} -1 & 0 & 0 & -1 \end{bmatrix} \widehat{\mathbf{x}}$$

$$\text{subject to} \qquad U^T \widetilde{\mathbf{x}} + \begin{bmatrix} -1 & 0 & 0 & 0 \\ -1 & 0 & 0 & 0 \\ & ... & & \\ -1 & 0 & 0 & 0 \end{bmatrix} \widehat{\mathbf{x}} \leq 0,$$

$$-U^T \widetilde{\mathbf{x}} + \begin{bmatrix} 0 & -1 & 0 & 0 \\ 0 & -1 & 0 & 0 \\ & ... & & \\ 0 & -1 & 0 & 0 \end{bmatrix} \widehat{\mathbf{x}} \leq 0,$$

$$\widetilde{V}^T U^T \widetilde{\mathbf{x}} + \begin{bmatrix} 0 & 1 & -1 & 0 \end{bmatrix} \widehat{\mathbf{x}} \leq \kappa',$$

$$\widetilde{W}^T U^T \widetilde{\mathbf{x}} + \begin{bmatrix} -1 & 0 & 1 & -1 \\ -1 & 0 & 1 & -1 \\ & ... & & \\ -1 & 0 & 1 & -1 \end{bmatrix} \widehat{\mathbf{x}} \leq -\kappa,$$

$$\widetilde{\mathbf{x}} \leq L,$$

$$-\widetilde{\mathbf{x}} \leq L,$$

$$\widehat{\mathbf{x}} \geq 0.$$

To further simplify the computations we change variables:

$$\mathbf{z} := \begin{bmatrix} 1 & 1 & ... & 1 & 0 & 0 & 0 \\ 0 & 1 & ... & 1 & 0 & 0 & 0 \\ & & ... & & & & \\ 0 & 0 & ... & 1 & 0 & 0 & 0 \\ 0 & 0 & ... & 0 & 1 & 0 & 0 \\ 0 & 0 & ... & 0 & 0 & 1 & 0 \\ 0 & 0 & ... & 0 & 0 & 0 & 1 \end{bmatrix} \mathbf{x}.$$

The above matrix has $n+2$ rows and $n+2$ columns. First $n-1$ rows and $n-1$ columns correspond to matrix U^T and the last three rows and columns correspond to identity matrix. Let's denote

$$\begin{aligned}\widetilde{\mathbf{z}} &:= \begin{bmatrix} z_1 & z_2 & ... & z_{n-1} \end{bmatrix}^T, \\ \widehat{\mathbf{z}} &:= \begin{bmatrix} z_n & z_{n+1} & z_{n+2} & z_{n+3} \end{bmatrix}^T.\end{aligned}$$

Then, the following equalities hold:

$$\begin{aligned}\widetilde{\mathbf{z}} &= U^T\widetilde{\mathbf{x}},\\ \widehat{\mathbf{z}} &= \widehat{\mathbf{x}}.\end{aligned}$$

Moreover, since

$$(U^T)^{-1} = \begin{bmatrix} 1 & -1 & 0 & ... & 0 & 0 \\ 0 & 1 & -1 & ... & 0 & 0 \\ & & & ... & & \\ 0 & 0 & 0 & ... & 1 & -1 \\ 0 & 0 & 0 & ... & 0 & 1 \end{bmatrix},$$

the following properties also hold:

$$\begin{aligned} x_i &= z_i - z_{i+1} \quad \text{for } i = 1, 2, ..., n-2, \\ x_{n-1} &= z_{n-1}. \end{aligned}$$

Using the above equalities, we can rewrite our optimization problem as

$$\begin{aligned}
\text{maximize} \quad & \widetilde{V}^T\widetilde{\mathbf{z}} + \begin{bmatrix} -1 & 0 & 0 & -1 \end{bmatrix} \widehat{\mathbf{z}} \\
\text{subject to} \quad & \widetilde{\mathbf{z}} + \begin{bmatrix} -1 & 0 & 0 & 0 \\ -1 & 0 & 0 & 0 \\ & ... & & \\ -1 & 0 & 0 & 0 \end{bmatrix} \widehat{\mathbf{z}} \leq 0, \\
& -\widetilde{\mathbf{z}} + \begin{bmatrix} 0 & -1 & 0 & 0 \\ 0 & -1 & 0 & 0 \\ & ... & & \\ 0 & -1 & 0 & 0 \end{bmatrix} \widehat{\mathbf{z}} \leq 0, \\
& \widetilde{V}^T\widetilde{\mathbf{z}} + \begin{bmatrix} 0 & 1 & -1 & 0 \end{bmatrix} \widehat{\mathbf{z}} \leq \kappa', \\
& \widetilde{W}^T\widetilde{\mathbf{z}} + \begin{bmatrix} -1 & 0 & 1 & -1 \\ -1 & 0 & 1 & -1 \\ & ... & & \\ -1 & 0 & 1 & -1 \end{bmatrix} \widehat{\mathbf{z}} \leq -\kappa, \\
& z_i - z_{i+1} \leq l_i \quad \text{for } i = 1, 2, ..., n-2, \\
& z_{n-1} \leq l_{n-1}, \\
& z_i - z_{i+1} \geq -l_i \quad \text{for } i = 1, 2, ..., n-2, \\
& z_{n-1} \geq -l_{n-1}, \\
& z_n, z_{n+1}, z_{n+2}, z_{n+3} \geq 0, \\
& \widetilde{\mathbf{z}} \in \mathbb{R}^{n-1}, \widehat{\mathbf{z}} \in \mathbb{R}^3.
\end{aligned}$$

Defining

$$V := \begin{bmatrix} \mu(s_1) & \mu(s_2) & ... & \mu(s_{n-1}) & -1 & 0 & 0 & -1 \end{bmatrix}^T,$$

$$V' := \begin{bmatrix}\mu(s_1) & \mu(s_2) & ... & \mu(s_{n-1}) & 0 & 1 & -1 & 0\end{bmatrix}^T,$$

$$W := \begin{bmatrix} \nu_1(s_1) & \nu_2(s_1) & ... & \nu_k(s_1) \\ \nu_1(s_2) & \nu_2(s_2) & ... & \nu_k(s_2) \\ & & ... & \\ \nu_1(s_{n-1}) & \nu_2(s_{n-1}) & ... & \nu_k(s_{n-1}) \\ -1 & -1 & ... & -1 \\ 0 & 0 & ... & 0 \\ 1 & 1 & ... & 1 \\ -1 & -1 & ... & -1 \end{bmatrix}$$

leads us to a final version of our optimization problem, namely

$$\begin{aligned} \text{maximize} \quad & V^T\mathbf{z} \\ \text{subject to} \quad & (V')^T\mathbf{z} \leq \kappa', \\ & W^T\mathbf{z} \leq -\kappa, \\ & z_i - z_n \leq 0 \quad \text{for } i = 1, 2, ..., n-1, \\ & -z_i - z_{n+1} \leq 0 \quad \text{for } i = 1, 2, ..., n-1, \\ & z_i - z_{i+1} \leq l_i \quad \text{for } i = 1, 2, ..., n-2, \\ & z_{n-1} \leq l_{n-1}, \\ & z_i - z_{i+1} \geq -l_i \quad \text{for } i = 1, 2, ..., n-2, \\ & z_{n-1} \geq -l_{n-1}, \\ & z_n, z_{n+1}, z_{n+2}, z_{n+3} \geq 0, \\ & \mathbf{z} \in \mathbb{R}^{n+3}. \end{aligned}$$

□

In the proof of Theorem 4, we mentioned that the transport plan for which the amount of signal transported between two auxiliary points is non-zero cannot be optimal. We are going to prove it now.

Theorem 5. *The transport plan γ such that $\gamma(\xi, \omega) > 0$ is not optimal in the sense of Wasserstein distance, i.e. there exists a transport plan γ' such that*

$$\int_{\mathbb{R}\cup\{\xi\}\times\mathbb{R}\cup\{\omega\}} \rho_2(x,y)d\gamma(x,y) > \int_{\mathbb{R}\cup\{\xi\}\times\mathbb{R}\cup\{\omega\}} \rho_2(x,y)d\gamma'(x,y),$$

where distance function ρ is defined as

$$\rho_2(x,y) = \begin{cases} |x-y| & \text{if } x, y \in \mathbb{R}, \\ \kappa & \text{if } x \in \mathbb{R}, y = \omega, \\ \kappa' & \text{if } x = \xi, y \in \mathbb{R}, \\ \kappa + \kappa' & \text{if } x = \xi, y = \omega. \end{cases}$$

Proof. Let's denote

$$\begin{aligned}\int_{x\in\mathbb{R}\cup\{\xi\}} d\gamma(x,\omega) &= p_0,\\ \int_{y\in\mathbb{R}\cup\{\omega\}} d\gamma(\xi,y) &= p_0'.\end{aligned}$$

Now let's define γ' in such a way that it is identical to γ for $(x,y)\in\mathbb{R}\times\mathbb{R}$, but:

$$\begin{aligned}\int_{x\in\mathbb{R}} d\gamma'(x,\omega) &= p_0,\\ \int_{y\in\mathbb{R}} d\gamma'(\xi,y) &= p_0'-\gamma(\xi,\omega),\\ \gamma'(\xi,\omega) &= 0.\end{aligned}$$

In other words, we construct γ' from γ by moving probability from $\{(\xi,\omega)\}$ to $\{(x,\omega): x\in\mathbb{R}\}$. Then we obtain:

$$\begin{aligned}\int_{\mathbb{R}\cup\{\xi\}\times\mathbb{R}\cup\{\omega\}} & \rho_2(x,y)d\gamma(x,y)\\ &= \int_{\mathbb{R}\times\mathbb{R}} |x-y|d\gamma(x,y)+\kappa(p_0-\gamma(\xi,\omega))+\kappa'(p_0'-\gamma(\xi,\omega))+(\kappa+\kappa')\gamma(\xi,\omega)\\ &= \int_{\mathbb{R}\times\mathbb{R}} |x-y|d\gamma(x,y)+\kappa p_0+\kappa' p_0'\\ &> \int_{\mathbb{R}\times\mathbb{R}} |x-y|d\gamma'(x,y)+\kappa p_0+\kappa'(p_0'-\gamma(\xi,\omega))\\ &= \int_{\mathbb{R}\cup\{\xi\}\times\mathbb{R}\cup\{\omega\}} \rho_2(x,y)d\gamma'(x,y).\end{aligned}$$

□

Bibliography

[1] Ciach, M. A., Miasojedow, B., Skoraczyński, G., Majewski, S., Startek, M., Valkenborg, D., & Gambin, A. (2020). Masserstein: Linear regression of mass spectra by optimal transport. *Rapid communications in mass spectrometry: RCM*, e8956. Advance online publication. https://doi.org/10.1002/rcm.8956.

[2] Santambrogio, F. (2015). *Optimal transport for applied mathematicians.* Springer.
